# Gaseous Synergistic Self‐Assembly and Arraying to Develop Bio‐Organic Photocapacitors for Neural Photostimulation

**DOI:** 10.1002/advs.202410471

**Published:** 2025-01-22

**Authors:** Xinyuan Fan, Yiming Tang, Jiahao Zhang, Kang Ma, Zhengyu Xu, Yuying Liu, Bin Xue, Yi Cao, Deqing Mei, Wei Wang, Guanghong Wei, Kai Tao

**Affiliations:** ^1^ State Key Laboratory of Fluid Power and Mechatronic Systems Key Laboratory of Advanced Manufacturing Technology of Zhejiang Province School of Mechanical Engineering Zhejiang University Hangzhou 310058 China; ^2^ Zhejiang‐Israel Joint Laboratory of Self‐Assembling Functional Materials ZJU‐Hangzhou Global Scientific and Technological Innovation Center Hangzhou 311215 China; ^3^ Zhejiang‐Ireland Joint Laboratory of Bio‐Organic Dielectrics & Devices School of Mechanical Engineering Zhejiang University Hangzhou 310058 China; ^4^ Department of Physics State Key Laboratory of Surface Physics Key Laboratory for Computational Physical Sciences (Ministry of Education) Fudan University Shanghai 200438 China; ^5^ Collaborative Innovation Center of Advanced Microstructures National Laboratory of Solid State Microstructure Department of Physics Nanjing University Nanjing 210093 China; ^6^ Jinan Microecological Biomedicine Shandong Laboratory Jinan 250021 China; ^7^ Institute for Brain Sciences Nanjing University Nanjing 210093 China; ^8^ Chemistry and Biomedicine innovation center Nanjing University Nanjing 210093 China

**Keywords:** bio‐photocapacitors, molecular manufacturing, neural photostimulation, physical vapor deposition, self‐assembly & arraying

## Abstract

Bioinspired supramolecular architectonics is attracting increasing interest due to their flexible organization and multifunctionality. However, state‐of‐the‐art bioinspired architectonics generally take place in solvent‐based circumstance, thus leading to achieving precise control over the self‐assembly remains challenging. Moreover, the intrinsic difficulty of ordering the bio‐organic self‐assemblies into stable large‐scale arrays in the liquid environment for engineering devices severely restricts their extensive applications. Herein, a gaseous organization strategy is proposed with the physical vapor deposition (PVD) technology, allowing the bio‐organic monomers not only self‐assemble into architectures well‐established from the solvent‐based approaches but morphologies distinct from those delivered from the liquid cases. Specifically, 9‐fluorenylmethyloxycarbonyl‐phenylalanine‐phenylalanine (Fmoc‐FF) self‐assembles into spheres with tailored dimensions in the gaseous environment rather than conventional nanofibers, due to the distinct organization mechanisms. Arraying of the spherical architectures can integrate their behaviors, thus endorsing the bio‐organic film the ability of programmable optoelectronic properties, which can be employed to design P‐N heterojunction‐based bio‐photocapacitors for non‐invasive and nongenetic neurostimulations. The findings demonstrate that the gaseous strategy may offer an alternative approach to achieve unprecedented bio‐organic superstructures, and allow ordering into large‐scale arrays for behavior integration, potentially paving the avenue of developing supramolecular devices and promoting the practical applications of bio‐organic architectonics.

## Graphical abstract

By eliminating solvophobic driving forces and solvent interference, a physical vapor deposition based gaseous organization strategy is delivered, allowing Fmoc‐FF self‐assemble into spheres with tailored dimensions. Furthermore, arraying of the spheres can integrate their behaviors, endorsing the bio‐organic film the ability of programmable optoelectronic properties, which can be employed to design P‐N heterojunction‐based bio‐photocapacitors for non‐invasive and nongenetic neurostimulations.

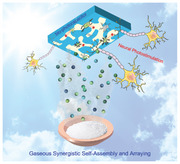

## 1 Introduction

Bio‐organic supramolecular architectures have gained increasing interest due to their inherent advantages of flexibility of design, simplicity of preparation, multifunctionality, and biocompatibility.^[^ ^1^, ^2^, ^3^, ^4^, ^5^ ^]^ However, state‐of‐the‐art bio‐organic architectonics generally occurs in the bulky liquids. Definitely, solution conditions such as solvent polarity, pH and electrolyte concentrations etc., significantly impact the self‐assembling thermodynamics and kinetics, and in most cases involve in the association procedures by supplying solvophobic interactions.^[^ ^6^, ^7^, ^8^ ^]^ On one hand, these effects may indorse molecular aligning and promote the formation of ordered supramolecular structures. However, the effects can also interfere with the organization, resulting in challenges to precisely control the morphologies as well as the properties of the bio‐organic architectures.^[^ ^9^, ^10^ ^]^ Therefore, it remains unclear what bio‐organicbio‐organic supramolecular architectonics would seem like in the absence of solvent influences. In addition, for practical applications, bio‐organic self‐assemblies need to be integrated into large‐scale arrays in terms of repeatability, processability and patterning, for devices machining and synergizing the features for signals I/O.^[^ ^11^, ^12^ ^]^ However, it is inherently challenging to order the bio‐organic superstructures into stable arrays at the presence of solvents, due to the dispersion effect which may lead to disassembly.^[^ ^11^ ^]^

PVD has been extensively employed to fabricate films for diverse advanced devices manufacturing. It can provide ultrahigh vacuum for inorganics or organic polymers to sublimate and deposit to form films of several nanometers in thickness,^[^ ^13^, ^14^, ^15^ ^]^ thus potentially excluding the solvent‐induced interference and endorsing alternative thermodynamic and kinetic pathways for enabling precise control over self‐assemblies. Especially, this strategy can allow the integration of the bio‐organic supramolecular architectures into large‐scale, programmable arrays with tailored mechanical and optoelectronic properties. In this regard, some reports demonstrate that bio‐organic building blocks, such as diphenylalanine and valine, could be utilized to achieve large‐scale arraying films with PVD.^[^ ^13^, ^16^ ^]^ This suggests the feasibility of engineering bio‐organic supramolecular architectonics in the gaseous way, as well as integration of the self‐assemblies into large‐scale arrays for device fabrications.^[^ ^11^, ^17^ ^]^

**Figure 1:**
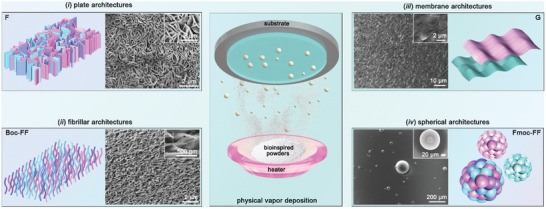
Gaseous organization strategy offers an alternative for bio‐organic self‐assembly as well as arraying. Middle panel: schematic cartoon depicting the bio‐organic gaseous organization with PVD technology. Left and right panels: diverse self‐assembling architectures, including i) plates, ii) fibers iii) membranes, and iv) spheres, either similar to or distinct from those gained in conventionally solvent situations, can be achieved. In addition, the supramolecular architectures can spontaneously arrange into large‐scale arraying films, allowing integration of the properties and endowing the feasibility to develop bio‐organic supramolecular devices. The insets display high‐magnification SEM images of the supramolecular architectures.

Herein, we report on bio‐organic gaseous organization using the PVD strategy. The absence of solvents can get rid of classical solvophobic interactions, thus offering alternative organization routines and resulting in various supramolecular architectures similar to or even distinct from those achieved in liquid cases. Subsequently, these bio‐organic supramolecular architectures could be ordered into large‐scale arraying films with integrated properties for advanced bio‐devices engineering (**Figure** **1**, middle panel). Our results exemplify that bio‐organic gaseous organization can be achieved, paving an alternative avenue of extending bio‐architectonics for practical applications in diverse biomedical and bio‐machine interfaces.

## 2 Result and Discussion

### 2.1 Gaseous Organization Offers Synergetic Bio‐Organic Self‐Assembly and Arraying

Briefly, when loaded on the evaporating dish and heated (See Experimental Section for details), the dry bio‐organic building block powders sublimated into gaseous states and deposited on a certain substrate, self‐assembling to form supramolecular architectures in a large scale. Scanning electron microscopy (SEM) characterizations demonstrated that different building blocks could self‐assemble into various morphologies (Table S1, Supporting Information). Specifically, vertically‐aligned plates (such as phenylalanine (F), dihydroxyphenylalanine, cyanuric acid and cordycepin etc.) and fibers (such as *tert*‐butyloxycarbonyl‐phenylalanine‐phenylalanine (Boc‐FF), FF, F(5fluoro)F and triphenyl‐triazine etc.) could be achieved (Figure 1i,ii; Table S1, Supporting Information), with the morphologies consistent with those self‐assembled from the bulky solutions. In addition, membranes could also be attained by some bio‐organic building units (such as glycine (G), Fmoc‐F, leucine‐valine, N‐[3‐(2‐furyl)acryloyl]‐FF and 1‐carboxyl‐tetraphenylethene etc.) (Figure 1iii; Table S1, Supporting Information). Especially, these supramolecular architectures could simultaneously accumulate into large‐scale, highly‐ordered arrays for potential devices engineering.

Intriguingly, in addition to the morphologies similar to the counterparts reported before, unexpected architectures distinct from those achieved from the solvent situations (especially in the aqueous solutions) could also be perceived through the gaseous organization strategy. Specifically, spherical morphologies rather than well‐established nanofibers in traditional liquid‐phase conditions (Figure S1, Supporting Information) were self‐assembled by Fmoc‐FF in the PVD situation (Table 1; Figure 1iv; Table S1, Supporting Information).^[^ ^18^ ^]^ This demonstrated that the gaseous organization strategy can offer an alternative routine for bio‐organic self‐assembly with no need of conventional solvophobic interactions as initial driving forces and simultaneously achieve arraying of the supramolecular architectures, thus expanding the architectural diversity and attaining large‐scale integration for developing bio‐organic supramolecular devices.^[^ ^11^ ^]^

**Figure 2:**
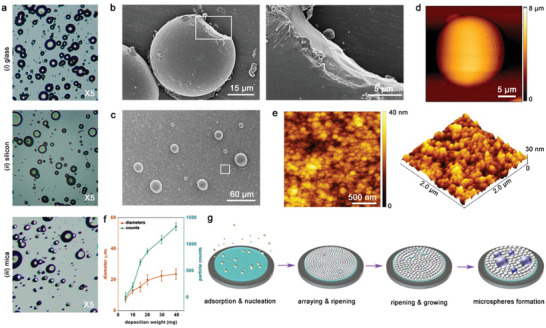
Architectural characterization of Fmoc‐FF spherical organization in the gaseous phase. a) Optical microscopy images of the bio‐organic supramolecular microspheres self‐assembled on diverse substrates. b) Left panel: SEM image showing the solid feature inside the microspheres. Right panel: high‐magnification SEM characterization of the captured rectangular framework in the left panel. c,d) SEM and AFM images depicting the co‐existence of large microspheres and a uniform film beneath. e) 2D (left) and 3D (right) viewpoint of high‐magnification AFM characterizations of the captured square area in (c) showing extensively‐collected nanoclusters inside the film. f) *ω*‐dependent diameter and amount evolution of the microspheres. g) Schematic cartoon depicting the hierarchical dynamics of Fmoc‐FF gaseous organization, experiencing several stages including adsorption, nucleation, ripening, and arraying.

The optical microscopy characterizations demonstrated that the spherical architectures could be self‐assembled on various substrates including glass, silicon, and mica (**Figure** **2**a), thus illustrating that the formation of the morphologies was dependent on the building blocks, irrespective of the substrate used. Solid configurations rather than hollow ones were observed in the fractured microspheres from SEM characterizations (Figure 2b), implying the rigid nature of the bio‐organic superstructures which will be discussed below. High‐magnification morphological characterizations demonstrated that a compact film existed on the substrate in addition to the microspheres (Figure 2c,d; Figure S2, Supporting Information). AFM measurements verified that the film was composed of extensively‐accumulated nanoclusters with a statistical diameter of 62.0 ± 21.3 nm (Figure 2e; Figure S3, Supporting Information), allowing us to hypothesize that the larger microspheres were derived from these oligomers. In this regard, the deposition weight (*ω*)‐dependent morphology and arraying evolution of Fmoc‐FF self‐assembly were investigated. The plots demonstrated that as ω increased, the microspheres gradually ripened from an initial size of 7.3 ± 3.5 µm at *ω* = 5 mg (Figure 2f; Figures S4–S8, Supporting Information). Nevertheless, the boosting rate retarded and the architectural diameter reached a pseudo plateau after ω was more than 30 mg, showing 23.6 ± 12.7 µm at *ω* = 40 mg (Figure 2f; Figure S9, Supporting Information). This suggested an antagonistic relationship between the curvature of the self‐assemblies and the agglomeration of the bio‐organic building blocks. Once a force balance was reached, the dimension of the spherical architecture kept consistent.^[^ ^19^ ^]^ In contrast, the quantity of the microspheres constantly increased, demonstrating that the arraying dimensions, including the film thickness and density, etc., could be adjusted by modulating *ω*.^[^ ^20^ ^]^ Based on these observations, we speculate that Fmoc‐FF gaseous self‐assembly experienced a hierarchical procedure.^[^ ^21^ ^]^ Specifically, the sublimated Fmoc‐FF molecules adsorbed on the substrate and organized into oligomeric nanoclusters, which simultaneously arrayed to form a large‐scale film. Followingly, the nanoclusters ripened into larger microspheres upon further collecting the building blocks until reaching forces equilibrium (Figure 2g).

### 2.2 Mechanism Underlying Fmoc‐FF Spherical Self‐Assembly

**Figure 3:**
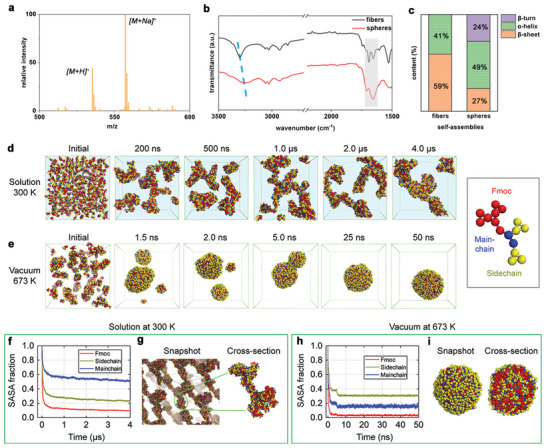
Characterization and process analysis of Fmoc‐FF gaseous self‐assembly. a) MS profile of the supramolecular microspheres, showing the native MW of Fmoc‐FF. b) FTIR spectra of the fibrillar and spherical architectures self‐assembled by Fmoc‐FF in the aqueous solution and gaseous phase situations, respectively. The blue dotted line represents the wavenumber shift of the O–H/N–H stretching vibrations between the two supramolecular morphologies. c) Secondary structures statistics of the fibrillar and spherical architectures, through deconvolution of the amide I region marked in grey color in (b). d,e) Snapshots at six‐time points showing Fmoc‐FF forming (d) fibril‐like aggregates in solution at 300 K and (e) spherical assemblies in the gas phase at 673 K. Note that the functional moieties in the Fmoc‐FF molecule are simplified and marked in different colors for clarity, as shown in the inset at the right panel. f) Time evolution of the SASA fraction for Fmoc, side‐chain, and main‐chain groups relative to their initial randomly dispersed states and g) Snapshot and cross‐sectional view of the fibril‐like aggregate in solution at 300 K. h) Time evolution of the SASA fraction for Fmoc, side‐chain, and main‐chain groups relative to their initial randomly dispersed states and i) Snapshot and cross‐sectional view of the spherical aggregates in gaseous phase.

It has been established that the unprotected linear aromatic dipeptides can transform to their cyclic counterparts by high‐temperature‐induced condensation reaction between the N‐terminal amino and C‐terminal carboxylic moieties in PVD situation, which may exacerbate the complicacy of the self‐assembling system.^[^ ^20^, ^22^ ^]^ In this regard, mass spectroscopy (MS) characterization demonstrated the intact molecular weight (MW) of Fmoc‐FF in the microspheres, showing two dominant peaks at *m/z* of 535.2 and 557.2 (**Figure** **3**a), corresponding to [M+H]^+^ and [M+Na]^+^, respectively. This confirms that rather than previously reported fragile bio‐organic building blocks, Fmoc‐FF molecules could remain stable during heating.^[^ ^23^ ^]^ In fact, X‐ray diffraction (XRD) characterization revealed the same interplanar spacing of 4.5 Å between the spherical architectures and nanofibers achieved from the conventional liquid case (Figure S10, Supporting Information), corresponding to the distance of adjacent aromatic entities in the self‐assemblies, thus suggesting that π–π stacking plays a critical role during Fmoc‐FF self‐assembly.^[^ ^24^, ^25^ ^]^

Nevertheless, FTIR characterizations demonstrated a significant bathochromic shift of the X–H stretching (X represents O or N atoms) vibrations from 3303 cm^−1^ in the nanofibers delivered in the aqueous solution to 3250 cm^−1^ in the spherical architectures, along with peak broadening (Figure 3b). This determined an extensive hydrogen bonding network existing in the spherical architectures,^[^ ^26^, ^27^ ^]^ thus illustrating that the self‐assembling secondary structures were distinct from those in the nanofibers. Therefore, deconvolution of the amide I region (1700–1600 cm^−1^) was then performed (Figure S11, Supporting Information). The results demonstrated that the well‐established nanofibers consisted of two type of secondary structures, β‐sheets, and α‐helices, with proportions of 59% and 41%, respectively (Figure 3c; Table S2, Supporting Information). This aligns with previous reports that Fmoc‐FF molecules organized into interlocked antiparallel β‐sheets or α‐helices based nanofibers in aqueous solutions, depending on the self‐assembly conditions.^[^ ^28^ ^]^ In contrast, in the spherical architectures, the profile was deconvoluted into three types of secondary structures: β‐sheets at 27%, α‐helices at 49%, and β‐turns at 24%, respectively (Figure 3c; Table S2, Supporting Information). This demonstrated that in the gaseous state, Fmoc‐FF molecules typically adopted kinked conformations rather than stretched ones for organization. Plausibly, high temperature could decrease the transformation activation energy and provoke the flexibility of the monomers, thus facilitating the conformational conversions for spatial intertwining. This is consistent with the self‐assembling spherical morphologies with solid interior, and ultrahigh mechanical rigidity of the arraying film discussed beneath. Therefore, it's speculated that bio‐organic self‐assembly can experience multifarious routines in the gaseous organization approach, enabling polymorphic supramolecular architectures with integrated features.^[^ ^29^ ^]^

In order to investigate the formation pathway and underlying mechanism of Fmoc‐FF spherical aggregation in the gaseous phase, molecular dynamics (MD) simulations were conducted. Specifically, two individual simulations were performed, starting from 600 randomly dispersed Fmoc‐FF molecules in a high‐temperature (673 and 500 K) vacuum environment to mimic the experimentally gaseous phase. As controls, two independent self‐assembly simulations of 600 Fmoc‐FF molecules in aqueous solutions at room temperature (300 K) were also investigated. In the aqueous solution case, the Fmoc‐FF molecules aggregated into rod‐like assemblies within 500 ns, which then merged and fused into large aspect‐ratio, branched architectures (Figure 3d; Figure S12, Supporting Information), exhibiting characteristic of nanofibrillar hydrogels and resembling nanostructures observed in previous MD simulations on other Fmoc‐containing dipeptides.^[^ ^30^, ^31^ ^]^ In contrast, in the vacuum system at 673 K, the Fmoc‐FF molecules organized more rapidly, achieving three clustered aggregates within 2 ns which soon merged into a single dumbbell‐shaped aggregate by 5 ns. Afterward, this aggregate evolved into a spherical morphology (Figure 3e), consistent with the experimental findings. In addition, similar results were obtained for the simulations at a lower temperature (500 K), albeit of relatively slower self‐assembly dynamics (Figure S12b, Supporting Information), thus confirming the accuracy of the theoretical findings. Subsequently, the fibrillar and spherical assemblies were characterized by comparing the solvent accessible surface area (SASA) of the Fmoc, side‐chain, and main‐chain moieties (marked in the inset of Figure 3d,e) at each time point to their SASA in the initial dispersed state. For the fibril‐like aggregates, the main‐chain group showed a significantly higher SASA fraction (≈0.5) than the Fmoc (≈0.1) and side‐chain ones (≈0.2), indicating that the main‐chain was the most solvent‐exposed group, leaving the other two counterparts tend to be buried inside the aggregate to compose the hydrophobic core (Figure 3f,g; Figure S12c,d, Supporting Information). This suggests that the hydrophobic interactions play a critical role during Fmoc‐FF aqueous self‐assembly, consistent with the state‐of‐the‐art concepts.^[^ ^18^, ^27^ ^]^ By contrast, in the spherical aggregation case, the side‐chain dominated the solvent exposure preference, with the highest SASA fraction at ≈0.3 compared to ≈0.2 for the main‐chain (Figure 3h,i; Figure S12e,f, Supporting Information). These findings demonstrate that in the gaseous phase, the absence of the solvophobic interactions allowed the hydrophobic moieties the availability of exposing outside the aggregates, thus accounting for the distinct self‐assembly mechanisms contrast to the routine in conventional solvent conditions.

**Figure 4:**
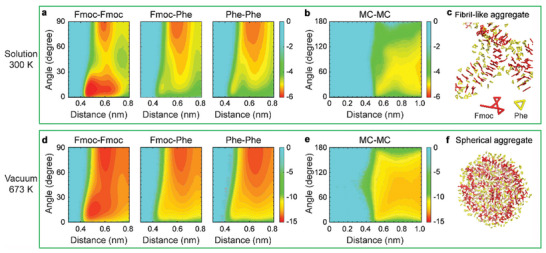
FES analysis of Fmoc‐FF self‐assembly. a–c) Fibril‐like aggregation in the solvents at 300 K. d–f) Spherical aggregation in the gaseous phase at 673 K. (a,d) FES of Fmoc‐Fmoc, Fmoc‐Phe, and Phe‐Phe aromatic stackings and (b,e) MC‐MC interactions as a function of centroid distance and angle between two aromatic rings/MCs for (top panel) fibril‐like and (lower panel) spherical aggregates, respectively. (c,f) Cross‐sectional views of the Fmoc‐FF fibril‐like and spherical aggregates, respectively, with Fmoc and Phe groups shown in stick representations.

Notably, the Fmoc group maintained the lowest SASA fraction among all groups in both cases, underscoring the necessity of π–π stacking among Fmoc moieties in driving the organization of Fmoc‐FF molecules.^[^ ^32^ ^]^ Therefore, the fibrillar and spherical self‐assembly were further investigated by establishing the free energy surface (FES) for π–π stacking interactions and main chain‐main chain (MC‐MC) interactions. At the presence of solvents, the FES for Fmoc‐Fmoc π–π stackings revealed two distinct basins: a deep basin (0.47 nm and 10°) indicative of strong parallel aromatic stacking, and a relatively shallow basin (0.58 nm and 90°) reflecting weaker T‐shaped aromatic stacking (**Figure** **4**a; Figure S13a, Supporting Information). While for the Fmoc‐Phe and Phe‐Phe interactions, only the T‐shaped basin was detected (Figure 4a; Figure S13a, Supporting Information). These observed stacking patterns are similar to those found in other Fmoc‐based peptides.^[^ ^30^, ^31^ ^]^ By contrast, only a shallow basin was detected in the FES for MC‐MC interactions (Figure 4b; Figure S13b, Supporting Information), suggesting a relatively minor role of these interactions in the fibrillization of Fmoc‐FF. These observations, together with visual inspections (Figure 4c; Figure S13c, Supporting Information), indicated that the solvent‐induced Fmoc‐FF self‐assembly was primarily driven by Fmoc‐Fmoc parallel π–π stacking, consistent with the SASA fraction analysis shown above, while the branching was facilitated by T‐shaped Fmoc‐Phe and Phe‐Phe aromatic interactions.

In the gaseous phase, the π–π stacking patterns between the aromatic groups resembled those in the solution state (Figure 4d,e; Figure S13d,e, Supporting Information), demonstrating the same types of driving forces in the two self‐assembly strategies. However, the energy basins in the gaseous phase were much deeper, indicating stronger π‐π stacking interactions contributed in the Fmoc‐FF spherical self‐assembly. Therefore, these findings provided molecular evidence for the XRD characterizations. Specifically, the Fmoc‐Fmoc π‐π stacking in gaseous phase did not have any angle preference, along with the cross‐section views of the configurations (Figure 4f; Figure S13f, Supporting Information), showing the isotropic packing features of the Fmoc‐Fmoc pair which was beneficial to the formation of spherical aggregations (Figure 4d; Figure S13d, Supporting Information). Additionally, the FES for MC‐MC interactions displayed a wide basin (Figure 4e; Figure S13e, Supporting Information), reminiscent of the formation of hydrogen bond network among the backbones within the spherical architectures and consistent with the FTIR results. These computational simulations demonstrated that in the gaseous organization case, the lack of solvophobic interactions induced distinct driving force contributions from those in the solvent‐based systems, thus accounting for the mechanism underlying alternative bio‐organic supramolecular architectonics along with delivering an avenue to study the effect of solvophobic interactions in peptide self‐assemblies.

### 2.3 Gaseous Organization‐Induced Arraying for Bio‐Organic Devices Development

**Figure 5:**
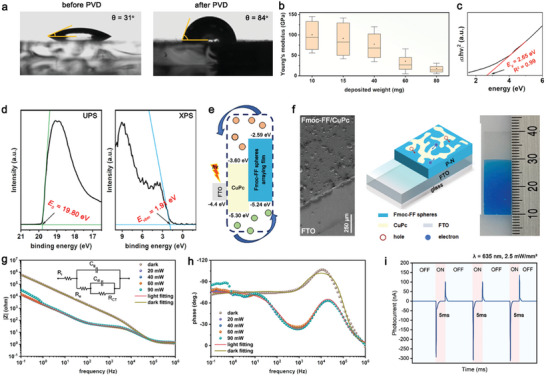
Fmoc‐FF spheres arraying film shows intrinsic N‐type semiconductivity to develop bio‐organic photocapacitors. a) Contact angle evolution before and after Fmoc‐FF deposition by PVD, showing a significant hydrophobicity enhancement of the substrate upon supramolecular spheres arraying film modification. b) Box diagram representing Young's moduli evolution versus *ω* of Fmoc‐FF, showing a gradual attenuation of the mechanical rigidity with self‐assemblies ripening. c) *(αhν)* ^[^ ^2^ ^]^ profile versus *hv* of Fmoc‐FF spheres arraying film derived from the UV–vis spectrum, showing a calculated *E_g_* of 2.65 eV by a linear fitting (R^2^ = 0.99). d) UPS (left panel) and XPS (right panel) spectra of the Fmoc‐FF spheres arraying film, showing *E_0_* of 19.80 eV and *E_vbm_* of 1.91 eV, respectively, by the intersection between the tangent and base lines. Note that the green and blue lines mark the baselines and tangent lines of the profiles. e) Schematic cartoon depicting the energy band gradient of the Fmoc‐FF/CuPc P‐N heterojunctions. f) Schematic diagram of the Fmoc‐FF/CuPc P‐N heterojunction‐based photocapacitors. The left and right panels show the SEM and photographic images of the device. g,h) Bode plots of (g) impedance and (h) phase frequency response under various irradiation conditions. The inset in (g) shows the equivalent circuit of the photocapacitor. i) Photocurrent curve of the photocapacitor under a pulsed irradiation (5ms, *λ* = 635 nm at 2.5 mW mm^−^^2^).

Arraying can significantly integrate and enhance the features of bio‐organic self‐assemblies, and especially, endow the bio‐organic supramolecular architectures the availability to be employed for engineering practical devices.^[^ ^11^, ^33^, ^34^, ^35^ ^]^ Regarding of this, spherical morphologies are much easier to order into highly‐ordered arrays compared to non‐spherical entities, due to their intrinsic advantage of being able to spontaneously organize into hexagonal lattices.^[^ ^36^ ^]^ While the gaseous organization strategy offers a synergetic approach for self‐assembly and large‐scale arraying.^[^ ^34^ ^]^ The characteristic application is to modify the solvophobicity of the adsorbed substrates.^[^ ^13^ ^]^ Herein, the contact angle of the hydrophilic glass wafer could be enhanced to 84° from native 31° upon modification by Fmoc‐FF spherical architectures arraying film (**Figure** **5**a), thus showing the feasibility of employing bio‐organic arrays to pattern for designing bio‐devices of functionality.

It has been established that aromatic peptide self‐assemblies possess high mechanical rigidity, due to the extensive vectoral non‐covalent interaction networks inside.^[^ ^21^, ^23^, ^37^ ^]^ Plausibly, arraying can allow integration and adjustment of the mechanical properties.^[^ ^38^ ^]^ Therefore, the Young's moduli of the bio‐organic spherical architectures arraying film were investigated by nanoindentation (Figure S14, Supporting Information).^[^ ^37^ ^]^ The results demonstrated that the Fmoc‐FF self‐assembling nanoclusters‐based film achieved at *ω* = 10 mg, presented an ultrahigh mechanical rigidity with a statistical Young's modulus of up to 100.5 ± 44.7 GPa (Figure 5b; Figure S15, Supporting Information), significantly higher than those of some inorganic metals such as Aluminium (68 GPa), gold (77.2 GPa) or calcium phosphate (83 GPa) etc.^[^ ^37^ ^]^ However, as the spherical architectures ripened (i.e., increasing *ω*), Young's moduli of the arraying film progressively attenuated, until 18.1 ± 12.9 GPa at *ω* = 80 mg (Figure 5b; Figures S16–S19, Supporting Information). Conceivably, the film roughness was low for smaller nanoclusters (Figure 2e), thus inducing a large arraying density and high integration. As *ω* increased, the film homogeneity gradually declined due to the assorted dimensional distributions, thus resulting in weakened integration and rigidity fading. These findings suggest the accessibility of delivering bio‐organic supramolecular arrays of programmable mechanical properties for diverse applications such as electromechanical coupling.^[^ ^38^ ^]^

In addition to the mechanical behaviors, aromatic short peptide self‐assemblies have been found to show intrinsic semiconductivity.^[^ ^39^ ^]^ Therefore, arraying of the bio‐organic supramolecular architectures may integrate their semiconductive features and facilitate development of peptide‐based optoelectronic devices.^[^ ^34^ ^]^

The optical bandgap of a semiconductor can be estimated from the Tauc plot (i.e., the curve of converted *(αhν)^r^* (*α*, *h*, and *ν* represent absorption coefficient, Planck constant and light frequency, respectively)) versus *hν* derived from the UV–vis absorption spectrum (Figure S20, Supporting Information), in which *r* = 2 and 0.5 for a direct bandgap and an indirect bandgap semiconductor).^[^ ^40^ ^]^ In this regard, the Tauc plot of the Fmoc‐FF spherical arraying film demonstrated a linear fitting when *r* = 2 (Figure 5c), validating the direct bandgap nature of the Fmoc‐FF self‐assembled spherical architectures. By extrapolating the fitting line to intercept on the X‐axis, the energy gap (*E_g_*) of the bio‐organic supramolecular architectures was determined to be 2.65 eV (Figure 5c).

Furthermore, the intersection points between the tangent and base lines from the ultraviolet photoelectron spectroscopy (UPS) and valence band X‐ray photoelectron spectroscopy (VB‐XPS) profiles determined the electron onset energy (*E_0_*) and valence band energy versus Fermi level (*E_vbm_*) to be 19.80 and 1.91 eV, respectively (Figure 5d; Figures S21 and S22, Supporting Information).^[^ ^41^ ^]^ Correspondingly, the work function (*WF*), the energy of the Fermi level relative to the vacuum level, was calculated to be 3.33 eV based on Equation (1):^[^ ^41^ ^]^

(1) $$WF=hv-\left(E_{0}-E_{vbm}\right)$$

where *hν* represents the width of the He I UPS spectra from the excitation energy (21.22 eV).

Followingly, the highest occupied molecular orbital (HOMO) and lowest unoccupied molecular orbital (LUMO) of the Fmoc‐FF self‐assembled spherical architectures were calculated to be 5.24 and 2.59 eV, respectively, by Equations (2) and (3):

(2) $$HOMO=WF+E_{vbm}$$

(3) $$LUMO=HOMO-E_{g}$$

Based on the relative locations between Fermi level and HOMO or LUMO, Fmoc‐FF spherical architectures could be termed as N‐type semiconductors.^[^ ^41^ ^]^

Upon co‐depositing copper(II) phthalocyanine (CuPc), a kind of P‐type organic semiconductor with HOMO and LUMO of 5.30 and 3.60 eV, respectively,^[^ ^42^ ^]^ with Fmoc‐FF as the acceptor layer using the gaseous organization strategy, a P‐N heterojunction could be fabricated (Figure 5e). Followingly, through introduction of F‐doped tin oxide modified glass slide (FTO) as the electrode, a bio‐organic ultrathin P‐N heterojunction‐based photocapacitor was engineered (Figure 5f), with the equivalent circuit shown in the inset of Figure 5g (where *R_e_* and *R_i_* signify the transfer resistance of electronic carriers and ions, respectively; the charge transfer resistance (*R_CT_*) is associated with the Faradaic process; the double layer capacitance (*C_dl_*) represents the semiconductor‐electrolyte interface; a complementary capacitance (*C_g_*) designates the geometric capacitance of the bio‐organic semiconductor layer).^[^ ^43^, ^44^ ^]^ The UV–vis absorption spectroscopy characterizations demonstrated that both the Fmoc‐FF spheres arraying film and CuPc film presented strong absorption between 600 and 700 nm (Figure S23, Supporting Information), endowing the feasibility of stimulating the bio‐organic P‐N heterojunction‐based photocapacitor with long‐wavelength irradiation.^[^ ^45^ ^]^ Therefore, the red light with a wavelength of 635 nm was subsequently employed to trigger the device.

The Bode plots of impedance (Figure 5g) and phase frequency response (Figure 5h) profiles by electrochemical impedance spectroscopy (EIS) analysis demonstrated that under illumination (*λ* = 635 nm, 2.5 mW mm^−^^2^), the P‐N heterojunctions could efficiently separate the photon‐induced electron‐hole pairs and transfer the excitons,^[^ ^43^, ^44^ ^]^ thus significantly reducing the resistances (from 423.6 to 36.6 Ω and 2.2 GΩ to 33.6 kΩ for *R_e_* and *R_CT_*, respectively) and enhancing the capacitance (from 67.0 nF to 1.3 µF and 2.3 to 55.0 µF for *C_g_* and *C_dl_*, respectively) of the optoelectronic device (Table S3, Supporting Information). In addition, the cyclic voltammetry (CV) measurements demonstrated that the photofaradaic process had a negligible impact on the resistance compared to the capacitive photocurrents (Figure S24, Supporting Information), implying the feasibility for photocapacitive applications of the bio‐organic device.^[^ ^13^ ^]^ Especially, upon exposed to a red light at λ = 635 nm sourced by a custom‐designed testing platform (Figure S25, Supporting Information), the bio‐organic photocapacitor was capable of generating photo‐excited electron‐hole pairs, which could be effectively separated for capacitive charge‐transfer by the inherent field in the P‐N junctions.^[^ ^46^ ^]^ Under pulsed irradiation of λ = 635 nm at 2.5 mW mm^−^^2^, the device exhibited sustainable signal outputs, showing a consistent photo‐responsive current at ≈250 nA (Figure 5i). As a control, the CuPc‐based one generated a much lower photocurrent (Figure S26, Supporting Information), thus verifying the efficiency for photoelectrons transformation of the heterojunctions. In addition, the FTIR spectra demonstrated no detectable changes of the film before and after illumination (Figure S27, Supporting Information), validating the capacitive nature of the photoresponses.

### 2.4 Neural Photo‐Stimulation of the Bio‐Organic Arraying‐Based Device

**Figure 6:**
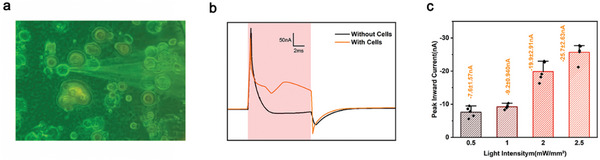
Light‐evoked neural stimulation using the bio‐organic arraying‐based photocapacitor. a) Photographic picture showing the cultured HT22 cells on the Fmoc‐FF/CuPc P‐N heterojunction‐based device during recording by patch clamping. b) Current traces recorded under “with cells” and “without cells” conditions. The red‐highlighted region indicates light stimulation (5 ms, λ = 635 nm, 2.5 mW mm^−^^2^). c) Peak inward current versus light intensity in the “with cells” condition.

The high‐efficiency light‐stimuli responses endow the bio‐organic photocapacitor the ability of being employed for applications in diverse bio‐photoelectronic fields.^[^ ^47^, ^48^ ^]^ In this regard, the cultured mouse hippocampal neuron cells (HT‐22) were seeded on the Fmoc‐FF/CuPc P‐N heterojunction‐based photocapacitor and incubated for 2 h until neuronal adhesion occurred. To assess the neuronal functionality, the well‐established patch clamp technique in voltage clamp mode was employed. Under irradiation at the red‐light pulse (λ = 635 nm at 2.5 mW mm^−^^2^), the membrane‐current of the neurons could be recorded (**Figure** **6**a).

Specifically, the electrophysiology of the neurons could be detected upon light exposure, showing a pronounced slow inward current with light stimulation (Figure 6b). As a control, negligible signals were recorded at the absence of neurons, thus validating the efficiency of the bio‐organic photocapacitor in motivating the neural activities. Especially, the neural cells exhibited modulatable discharge amplitudes upon tuning the exposure durations, showing the measured peak inward currents of 7.6 ± 1.6, 9.2 ± 0.9, 19.9 ± 2.9, and 25.7 ± 2.6 nA at light intensities of 0.5, 1.0, 2.0, and 2.5 mW mm^−^^2^, respectively (Figure 6c). These results exemplify the promising application prospect of the bio‐organic gaseous self‐assembly and arraying strategy to develop large‐scale, thin‐film‐based bio‐devices for non‐genetically manipulating neural behaviors, such as non‐invasive neural stimuli and remote bio‐optoelectronic interfaces.^[^ ^49^, ^50^ ^]^

## 3 Conclusion

The gaseous self‐assembly and arraying were simultaneously achieved for bio‐organic supramolecular architectonics by the PVD technology. The exclusion of solvophobic driving forces and solvent interference induced an alternative organizing routine for bio‐building units, resulting in spherical architectures rather than conventional nanofibrils for Fmoc‐FF self‐assembly. Especially, the architectural dimensions could be programmably tuned from nanoscale nanoclusters to larger microspheres, just easily by controlling the deposition weight. Characterizations demonstrated that different self‐assembling secondary structures were employed in the spherical architectures compared to the aqueous nanofibrils, thus accounting for the mechanism underlying distinct morphologies along with properties. Especially, the advantage of arraying into large‐scale films synergizing with self‐assembly in the gaseous organization approach endowed the availability of integrating the properties for developing bio‐organic devices. Specifically, Fmoc‐FF spheres arraying film presented N‐type semiconductivity, thus allowing to be employed to engineer bio‐organic P‐N heterojunction‐based photocapacitors for non‐invasive neural photostimulation.

In brief, the gaseous organization situates an alternative to achieve unprecedented structures for bio‐organic architectonics and allows ordering into large‐scale arrays. This may pave the way of integrating the functionalities and developing bio‐organic supramolecular devices, thus promoting the practical applications of bio‐organic self‐assembly in the realm of bio‐machine interface and biomedical engineering fields.

## 4 Experimental Section

### Materials

The bio‐organic materials with purities of > 95%, as listed in Table S1 (Supporting Information), and copper(II) phthalocyanine (CuPc) were purchased from GL Biochem (Shanghai, China), Sigma–Aldrich (Shanghai, China) or Bachem (Bubendorf, Switzerland), respectively. All the materials were used as received without further purification. Organic solvents (hexafluoroisopropanol (HFIP), acetone, and toluene) were purchased from Sinopharm Chemical Reagent Co., Ltd. Ultrapure water with a minimum resistivity of 18.2 MΩ cm was processed using a UP PLUS‐L purification system (Lichen, China).

### Bio‐Organic Gaseous Self‐Assembly and Arraying

The bio‐organic gaseous organization was performed with the custom‐designed PVD system as reported before.^[^ ^14^ ^]^ The building block powders with varied deposition weight (*ω*) were loaded on the precursor sample‐powder holder. The distance between the holder and the substrate was set at 1.6 cm. To ensure uniform deposition, the substrates were mounted on a rotating plate (10 rpm). The substrates were affixed on a rotating plate, in order to ensure homogeneous adsorption and organization. Upon heating the sample holder under an ultralow pressure of < 10^−5^ Pa, the bio‐organic building blocks sublimated to gaseous state and adsorbed on the substrate for organization. The characteristic deposition parameters (temperature routine, heating rate, holding time at each temperature interval) is listed below, to ensure reproducibility and uniform morphology of the spherical self‐assemblies. (**Table** **1**)

**Table 1:** PVD Parameters employed for gaseous synergistic self‐assembly and arraying.

Parameters	Values
Weight [ω]	As required
Distance between holder and substrate	1.6 cm
Chamber pressure	< 10^−5^ Pa
Temperature region [°C]	RT–260, 260–338, 338–400
Heating rate [°C min^−1^]	20, 15, 10
Holding time [min]	2, 2, 10

### Fmoc‐FF Aqueous Self‐Assembly

The Fmoc‐FF self‐assembled supramolecular nanofibers were prepared via the conventional solvent‐switch method.^[^ ^18^, ^21^ ^]^ The accurately‐weighed dipeptide powder was added into HFIP and stirred magnetically until fully dissolved. This led to a stock solution with a concentration of 50.0 mg mL^−1^. Thereafter, the solution was diluted with water at a ratio of 1:4, resulting in sample solutions with the Fmoc‐FF concentration at 10.0 mg mL^−1^. The solution rapidly transformed into a turbid and viscous substance, validating that the self‐assembly was completed.

### Characterizations

The determination of the surface morphologies and phase structures was conducted with SEM (Hitachi SU‐8010) and XRD (Shimadzu XRD‐6000). Scanning parameters for XRD experiment were set as a speed of 4° min^−1^, with Cu‐Kα radiation at λ = 1.5418 Å, and a scan range between 10° and 80°. AFM images were captured with a Bruker (Veeco)‐MultiMode instrument, at a scanning size of 2 × 2 µm. MS trials were implemented using a Bruker matrix, aided by a laser analytical ionization time‐of‐flight series MS (MALDITOFTOF+ImagePrep). FTIR assessments were facilitated with a Thermo Scientific Nicolet iS10 machine. The assessment of the contact angles was performed on a video‐based contact angle measurement device (OCA, Dataphysics OCA 20). The examination of the static contact angles of the films was performed via the sessile drop method, anchored on the principles of optical video recording. UV–vis spectroscopic experimentation was carried out at room temperature, employing a Shimadzu UV‐2600 spectrometer. Thermo Scientific Escalab 250Xi was used to perform XPS and UPS, featuring an Al Kα X‐ray source of 1486.6 eV for XPS and He I of 21.2 eV for UPS.

### MD Simulations

The MD simulations were carried out using the GROMACS package^[^ ^51^ ^]^ (version 2022.6) in combination with the MARTINI coarse‐grained (CG) model (version 2.2).^[^ ^52^ ^]^ The force field parameters for the FF dipeptide were taken from the MARTINI protein model,^[^ ^53^ ^]^ and the Fmoc group was described using CG models developed by ourselves.^[^ ^54^ ^]^ For the gaseous phase simulations, 600 uncharged Fmoc‐FF molecules were randomly placed in a 20 × 20 × 20 nm^3^ cubic box to generate the initial state. For the solution phase simulations, the C‐terminal main‐chain bead of the Fmoc‐FF molecule was assigned a negative charge, and 600 Fmoc‐FF molecules were randomly inserted into a 20 × 20 × 20 nm^3^ box containing 63 000 water beads. Electrostatic interactions were treated using the Particle Mesh Ewald (PME) method^[^ ^55^ ^]^ with a real space cut‐off of 1.2 nm. The vdW interactions were calculated using a cut‐off of 1.2 nm. The solute and solvent were separately coupled to external temperature baths of 300 K (for solution phase simulations) and 673/500 K (for gaseous phase simulations) using a velocity rescaling method.^[^ ^56^ ^]^ For the solution phase simulation, all molecules were also coupled to a pressure bath using the Parrinello‐Rahman method.^[^ ^57^ ^]^ The neighbor‐list was updated every 10 steps with a cut‐off distance of 1.2 nm using a Verlet buffer.

### Trajectory Analysis

For the calculation of SASA, the van der Waals radius was set as 0.264 and 0.23 nm for all regular beads (including the water bead) and small beads, respectively.^[^ ^52^ ^]^ The SASA fraction of each group (Fmoc, main‐chain, and side‐chain) was defined as the percentage of the SASA of that group relative to its SASA in the initial randomly dispersed state. The angle between two aromatic rings referred to the angle between the normal vectors of the two rings. If the angle was larger than 90°, the supplementary angle was used. The main‐chain of each molecule was treated as vector starting from its N‐terminal bead to its C‐terminal bead. The angle between two main‐chains (0°and 180°) referred to the angle between there corresponding vectors. The 2‐D free energy surfaces were constructed using the relation ‐*RT*ln[*P*(angle, centroid distance)], where *P*(angle, centroid distance) was the probability of a stacking pattern to have a certain value of angle and centroid distance. The data in the last 2.0 µs of the solution phase simulations, and the last 25/100 ns of the 673/500 K gaseous phase simulations were used to construct the free energy surface. Trajectory visualization and graphical structure analysis were performed using the PyMOL software suite.^[^ ^58^ ^]^

### Measurement of Young's Modulus

AFM experiments were performed using a commercial AFM (JPK, Nanowizard II, Berlin, Germany) with silica cantilevers (SSS‐SeIHR‐50, Nanosensor, tip radius: 2–20 nm, spring constant: 10–130 N m^−1^, frequency: 96–175 kHz). The maximum loading force was set at 500 nN, and all experiments were conducted at room temperature. The Fmoc‐FF self‐assembled spherical films were placed on the specimen stage, and the cantilever moved across the film at a constant speed of 15 µm s^−1^ under optical microscope guidance. Force curves were analyzed using a custom‐written Igor Pro 6.12 program, with manual fitting performed for accuracy. Each approaching force‐deformation curve was fitted within 20 nm of the contact point using the Hertz model (4), assuming a Poisson ratio (ν) of 0.3. The Young's modulus (E) was calculated as:

(4) $$F\;\left(h\right)=\frac{2}{\pi }\;\tan\alpha \frac{E_{GW}}{1-v_{GW}^{2}}h^{2}$$

where *F* is the applied force, *h* is the indentation depth, *α* is the half angle of the tip, *E* is the Young's modulus, and *ν* is the Poisson ratio. To ensure reliability, measurements were performed on 5–8 randomly selected regions (2 × 2 µm and 128 × 128 pixels) per film, using three cantilevers of the same type to exclude tip‐to‐tip dependency.

### Energy Band Calculations

The optical bandgap (*E_g_*) of the Fmoc‐FF self‐assembled spheres arraying film was estimated from the Tauc plot, which represents the curve of the converted *(αhν) ^r^* versus *hv* from the UV–vis absorption spectrum. Herein, *α*, *h*, and *v* represent the absorption coefficient, Planck constant, and light frequency, respectively, and *r = 2* for a direct bandgap material and *r = 1/2* for an indirect bandgap material. Figure 5c in the main text demonstrates a good linear fitting when *r = 2*, substantiating that the Fmoc‐FF film ensued to be a direct bandgap semiconductor. In contrast, no good linear fitting was obtained when *r = 1/2*. Based on the valence band X‐ray photoelectron spectrum (VB‐XPS), the linear part of the rendered graph near 0 eV was extrapolated to intersect with the horizontal extension line, and the intersection point was the valence band energy (*E_wbm_*) of the relative Fermi level. The work function (*WF*) was calculated by subtracting the width of the spectrum of the He I UPS from the excitation energy (21.22 eV), which is the energy of the Fermi level relative to the vacuum level. Correspondingly, the HOMO and LUMO could be calculated by E*_g_*, *E_wbm_*, and *WF*.

### Fabrication of Fmoc‐FF/CuPc P‐N Heterojunction‐Based Photocapacitors

The FTO modified glass slide was cleaned by sequential sonication in toluene, acetone, ethanol, and deionized water. All samples underwent UV/ozone treatment for 15 min thereafter. Stencil shadow masking was used in patterning the organic semiconductor layer. The FTO‐glass with a dimension of 1 cm × 3 cm, showing a mask of 1 cm at the edge, allowed the P‐type CuPc and N‐type Fmoc‐FF to be steamed by PVD on the remaining 1 cm × 2 cm consecutively, to finish the fabrication of the P‐N heterojunction photocapacitor.

### Electrochemical Characterizations of the P‐N Heterojunction‐Based Photocapacitors

The electrochemical impedance spectroscopy (EIS) and cyclic voltammetry (CV) curves were derived from the electrochemical characterization procedures. The chronopotentiometry and chronoamperometry measurements were conducted using a CHI660E electrochemical workstation, employing a three‐electrode system configured with an Ag/AgCl reference electrode, a platinum wire counter electrode, and the photocapacitor as the working electrode, all immersed in a 1.0 m KCl solution. The impedance analyzer operated over a frequency range from 0.1 Hz to 1 × 10^6^ Hz, while the CV voltage scan spanned from −1.2 to 0.5 V.

### Photoelectric Tests of the Bio‐Organic P‐N Heterojunction Photocapacitor

The photoelectric measurements of the Fmoc‐FF/CuPc P‐N heterojunction photocapacitor were conducted employing the Keithley 2400 apparatus. A fixed‐wavelength (635 nm) laser emitter, adjustable in power, served as the source of the optical stimulus. The PM400 optical power meter by THORLABS was utilized to standardize its power to four distinct levels, precisely 0.5, 1.0, 2.0, and 2.5 mW mm^−^^2^. These served as a comparative reference for regulating the light source's intensity. Experiments were carried out in a dark Faraday cage. A sample stand with a central hole of 4 cm in diameter was positioned on the Z‐axis shifter. Its elevation facilitated the placement of a laser light source underneath such that the light could traverse the hole directly from bottom‐up, impacting the photocapacitor to be stationed on the stand. One probe was deployed to create contact with the transparent conductor back electrode of the device (bare FTO) and another probe was utilized to establish contact with the P‐N joint. By assiduously observing the image on the overhead optical microscope, the Z‐axis shifter was manipulated to ensure that both probes precisely touched the device. The two probes were connected with the positive and negative electrodes, respectively, on the source meter to record the photocurrent from the device or to apply a bias voltage.

### Electrophysiological Experiment Based on Patch‐Clamp Technique

The cultured HT‐22 cells were seeded on the Fmoc‐FF/CuPc P‐N heterojunction‐based photocapacitor and incubated for 2 h until neurons settled. The device was transferred to the recording chamber and superfused (≈3 mL min^−1^) with ACSF containing (in mm): 135.0 NaCl, 5.0 KCl, 1.8 CaCl_2_, 1.0 MgCl_2_, 10.0 HEPES, and 10.0 glucose (pH 7.4 adjusted by NaOH). Cells were visualized with infrared optics using an upright microscope equipped with a 40× lens. For cell‐attached recording, pipettes with resistances of 6–8 MΩ were pulled from borosilicate glass capillaries and containing (in mm): 135.0 K‐gluconate, 5.0 KCl, 0.5 CaCl_2_, 10.0 HEPES, 2.0 Mg‐ATP, 0.1 GTP, and 5.0 EGTA, 300.0 mOsm (pH adjusted to 7.3 with KOH). To record red light‐evoked responses, light at λ = 635 nm was turned on for 5, 10, and 20 s to directly illuminate the patched cell. All signals were acquired with a MultiClamp 200B amplifier (Molecular Devices), filtered at 1 kHz, and sampled at 5 kHz with a Digidata 1322 interface using Clampex 10.2 (Molecular Devices). The number of cells varied with each individual experiment and was indicated in the respective figure legends. Data were presented as mean ± standard error of the mean.

## Conflict of Interest

The authors declare no conflict of interest.

## Supporting information



Supporting Information

## Data Availability

The data that support the findings of this study are available from the corresponding author upon reasonable request.
