# Supplementary material for: Gaseous Synergistic Self‐Assembly and Arraying to Develop Bio‐Organic Photocapacitors for Neural Photostimulation
Source: Adv Sci (Weinh). 2025 Jan 22;12(15):2410471. doi: 10.1002/advs.202410471 (PMC12005802; doi:10.1002/advs.202410471)
Supplement: Supplementary file 1 — Supporting Information [file ADVS-12-2410471-s001.docx]

Supporting Information

Gaseous Synergistic Self-Assembly and Arraying to Develop Bio-Organic Photocapacitors for Neural Photostimulation

Xinyuan Fan^†^, Yiming Tang^†^, Jiahao Zhang, Kang Ma, Zhengyu Xu, Yuying Liu, Bin Xue, Yi Cao, Deqing Mei, Wei Wang, Guanghong Wei ^*^, Kai Tao^*^

**Table S1** Diverse supramolecular architectures achieved in large-scale by bioinspired building blocks through the gaseous organization strategy

| **architectural morphology** | **building unit** | **chemical structure** | **SEM image** |
| --- | --- | --- | --- |
| membranes | 9-fluorenylmethyloxycarbonyl-phenylalanine  (Fmoc-F) | 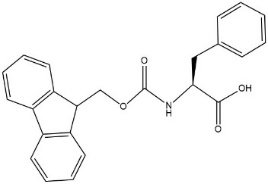 | 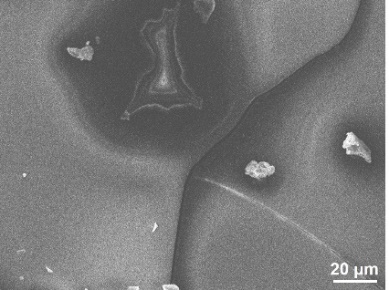 |
|  | glycine  (G) | 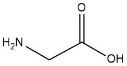 | 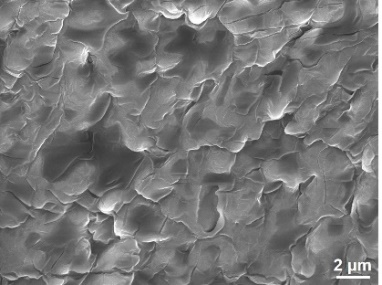 |
|  | N-[3-(2-furyl)acryloyl]- diphenylalanine  (FA-FF) | 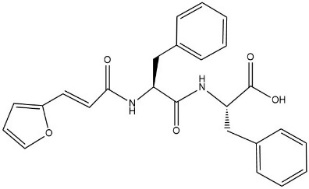 | 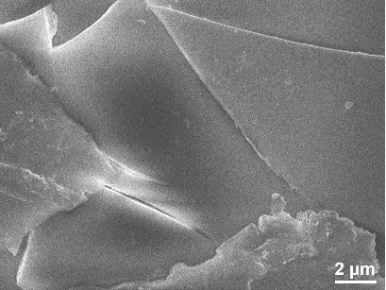 |
|  | leucine-valine  (LV) | 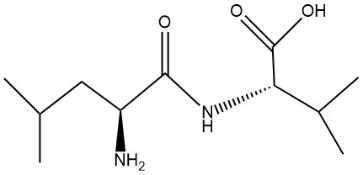 | 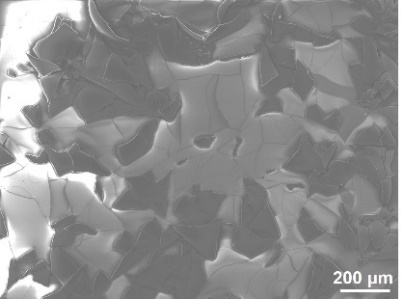 |
|  | 1-carboxyl-tetraphenylethene | 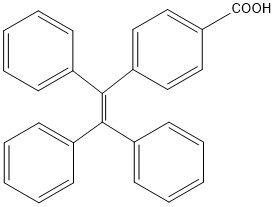 | 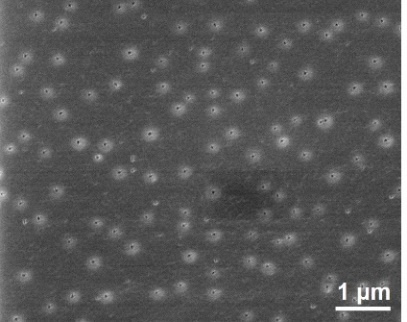 |
| plates | phenylalanine  (F) | 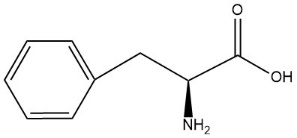 | 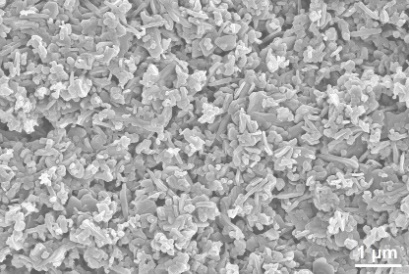 |
|  | dihydroxyphenylalanine  (DOPA) | 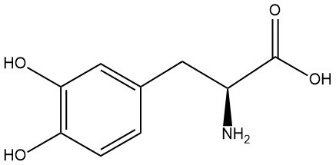 | 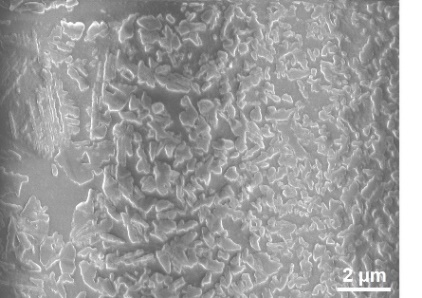 |
|  | cyanuric acid | 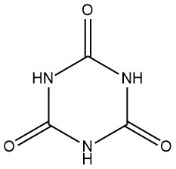 | 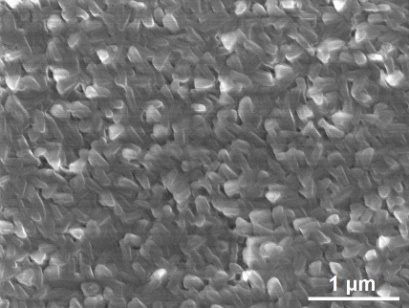 |
|  | cordycepin | 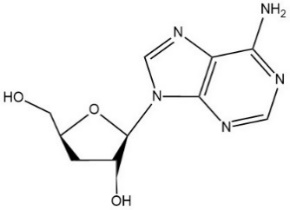 | 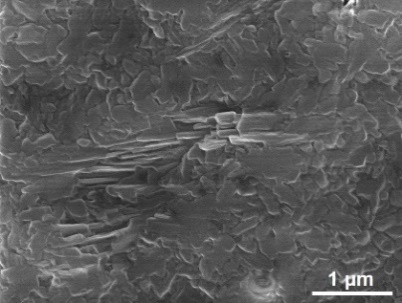 |
| fibers | diphenylalanine  (FF) | 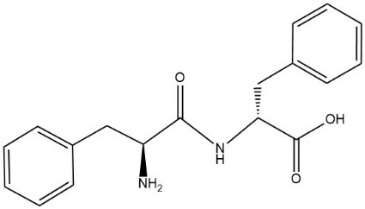 | 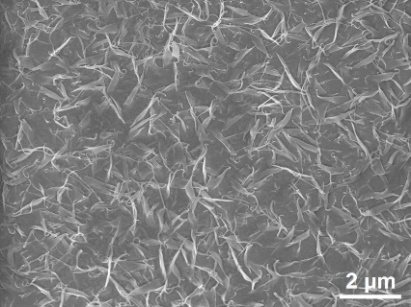 |
|  | *tert*-butyloxycarbonyl-phenylalanine-phenylalanine  (Boc-FF) | 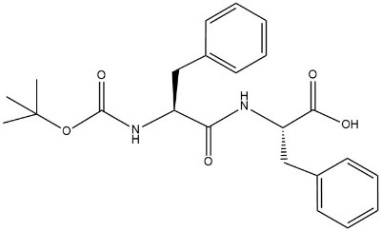 | 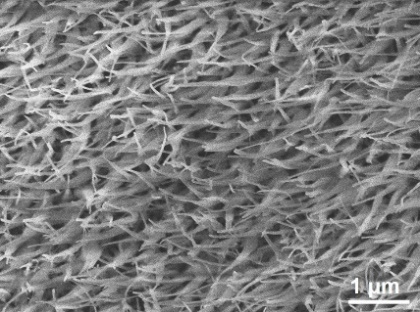 |
|  | phenylalanine-(5-fluoro)phenylalanine  [F(5-fluoro)F] | 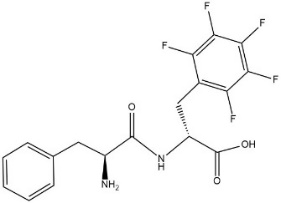 | 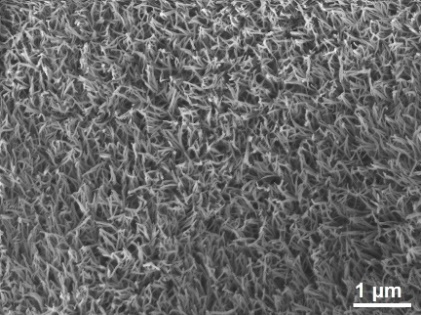 |
|  | triphenyl-triazine | 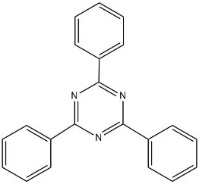 | 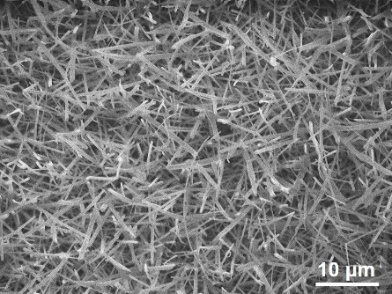 |
| spheres | 9-fluorenylmethyloxycarbonyl-diphenylalanine  (Fmoc-FF) | 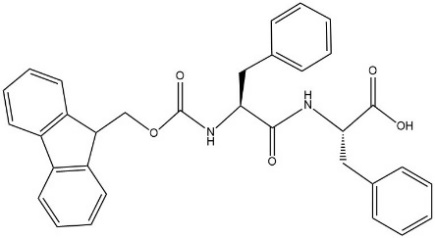 | 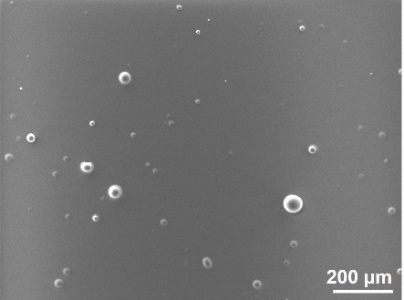 |


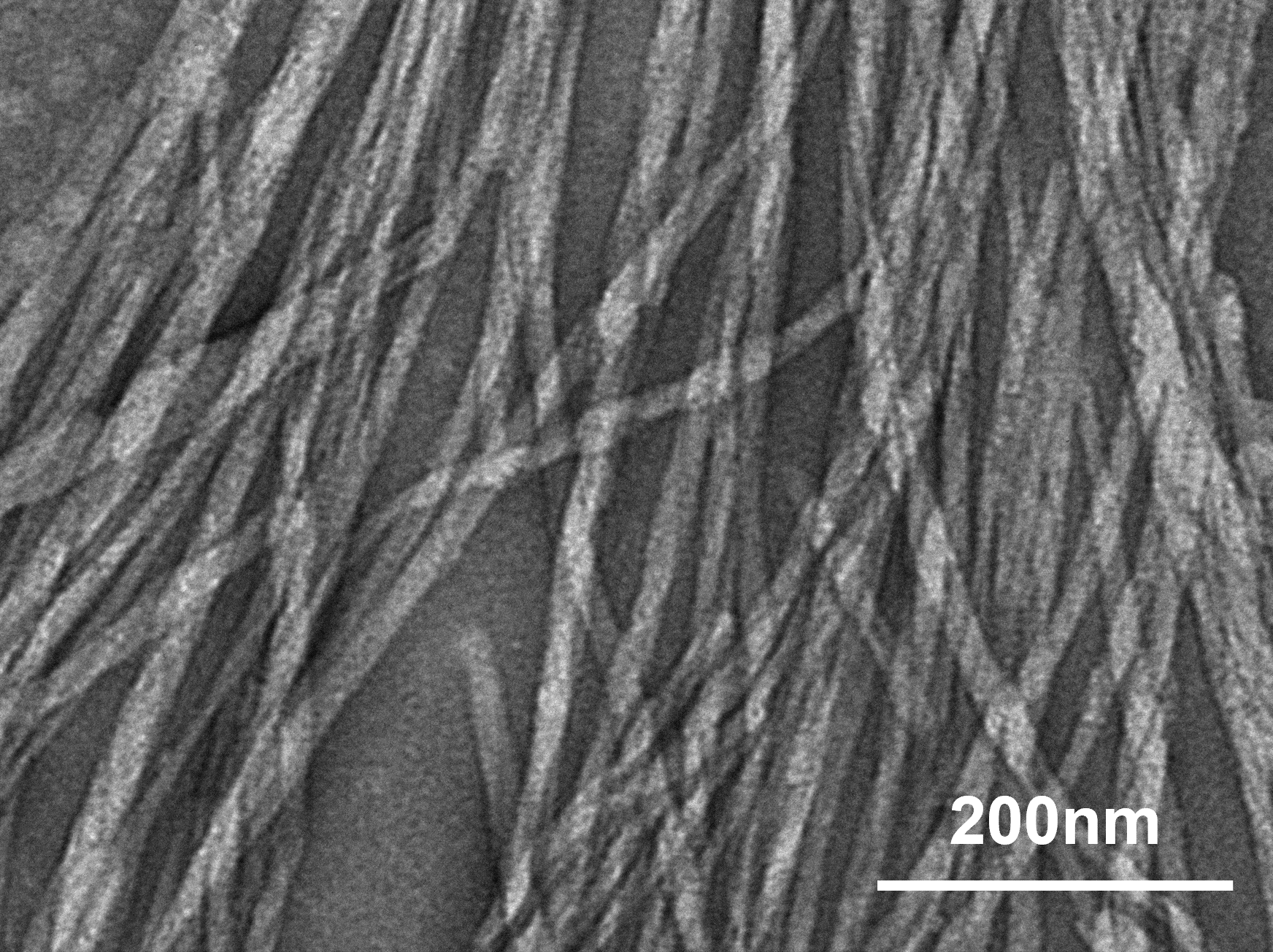


**Figure S1** SEM image of the Fmoc-FF self-assemblied nanofibers in the liquid phase (aquesou solution).


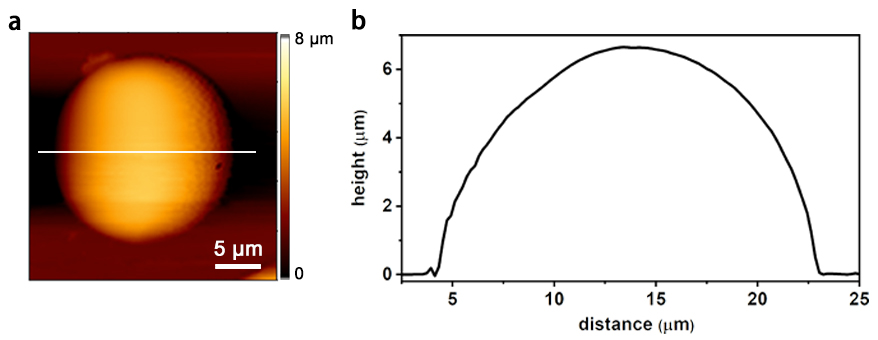


**Figure S2** Topological characterization of the Fmoc-FF self-assembled sphere by AFM. **a**, AFM image of the microsphere shown in Figure 2d in the main text. **b**, Height profile along the white line shown in (**a**).

The results demonstrate that the height of the self-assembled microsphere could reach up to approximately 7 μm.





**Figure S3** Statistical diameter distribution of the oligomeric nanoclusters self-assembled by Fmoc-FF from AFM, showing an average of 62.0 ± 21.3 nm. For accuracy, no less than 500 records were counted for average.


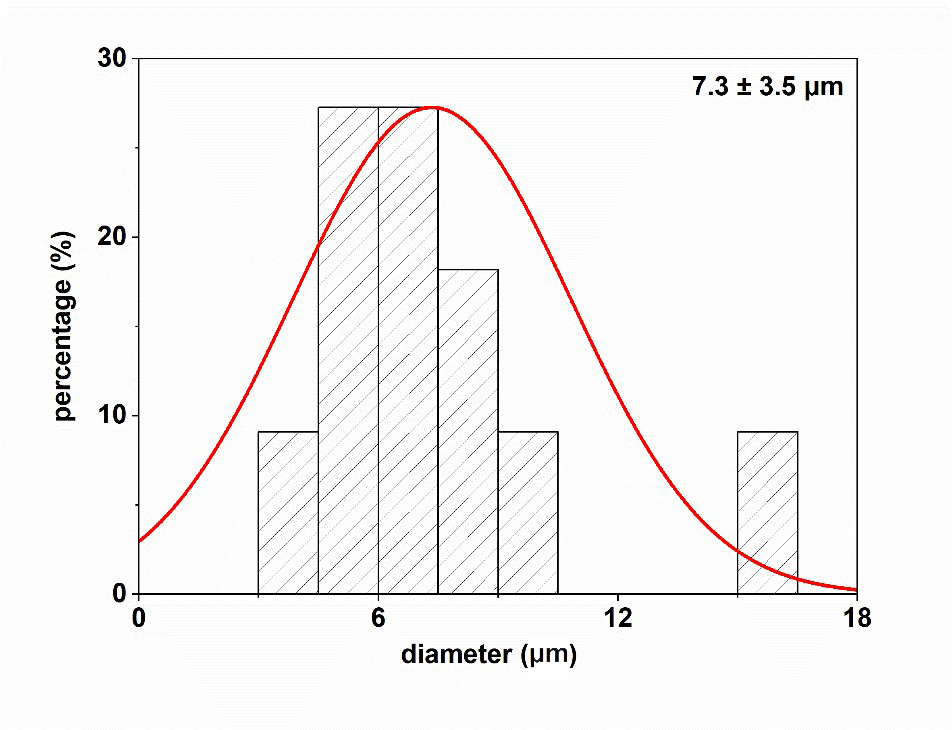


**Figure S4** Statistical diameter distribution of the self-assembled microspheres achieved upon *ω* = 5 mg by SEM, showing a statistical average of 7.3 ± 3.5 μm. For accuracy, no less than 100 records were counted for average.


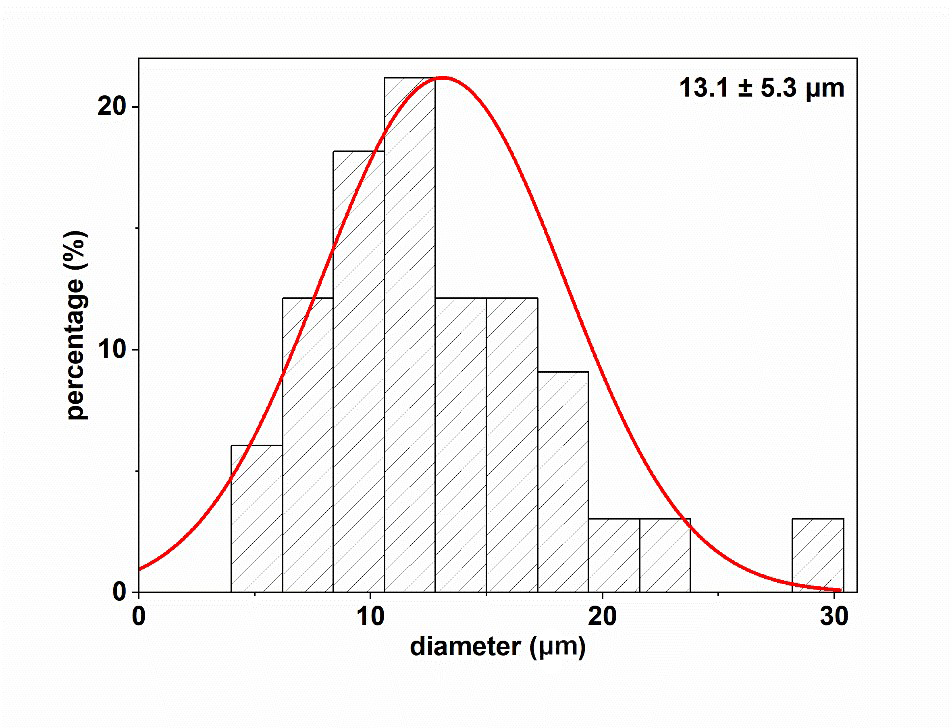


**Figure S5** Statistical diameter distribution of the microspheres achieved upon *ω* = 10 mg by SEM, showing a statistical average of 13.1 ± 5.3 μm. For accuracy, no less than 300 records were counted for average.


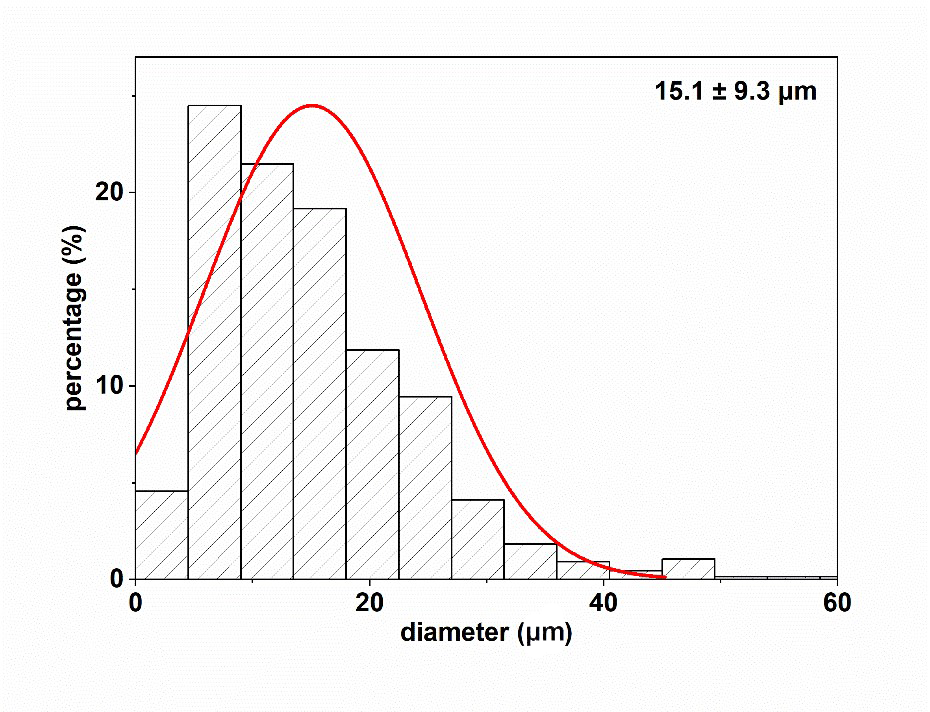


**Figure S6** Statistical diameter distribution of the microspheres achieved upon *ω* = 15 mg by SEM, showing a statistical average of 15.1 ± 9.3 μm. For accuracy, no less than 800 records were counted for average.


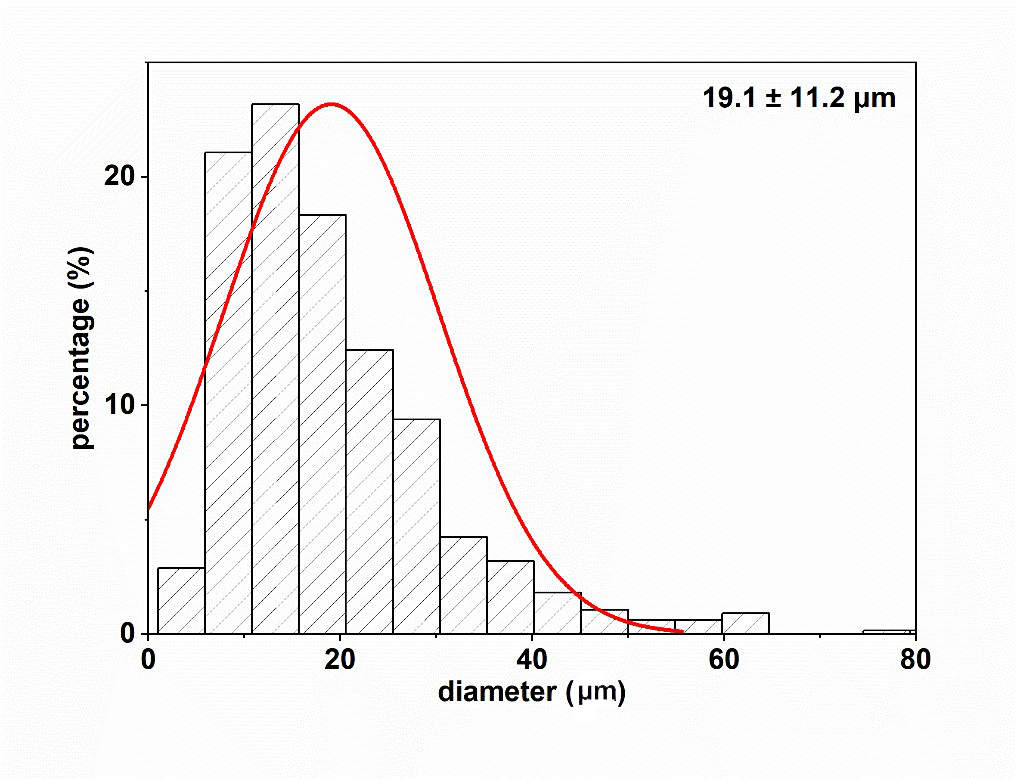


**Figure S7** Statistical diameter distribution of the microspheres achieved upon *ω* = 20 mg by SEM, showing a statistical average of 19.1 ± 11.2 μm. For accuracy, no less than 600 records were counted for average.


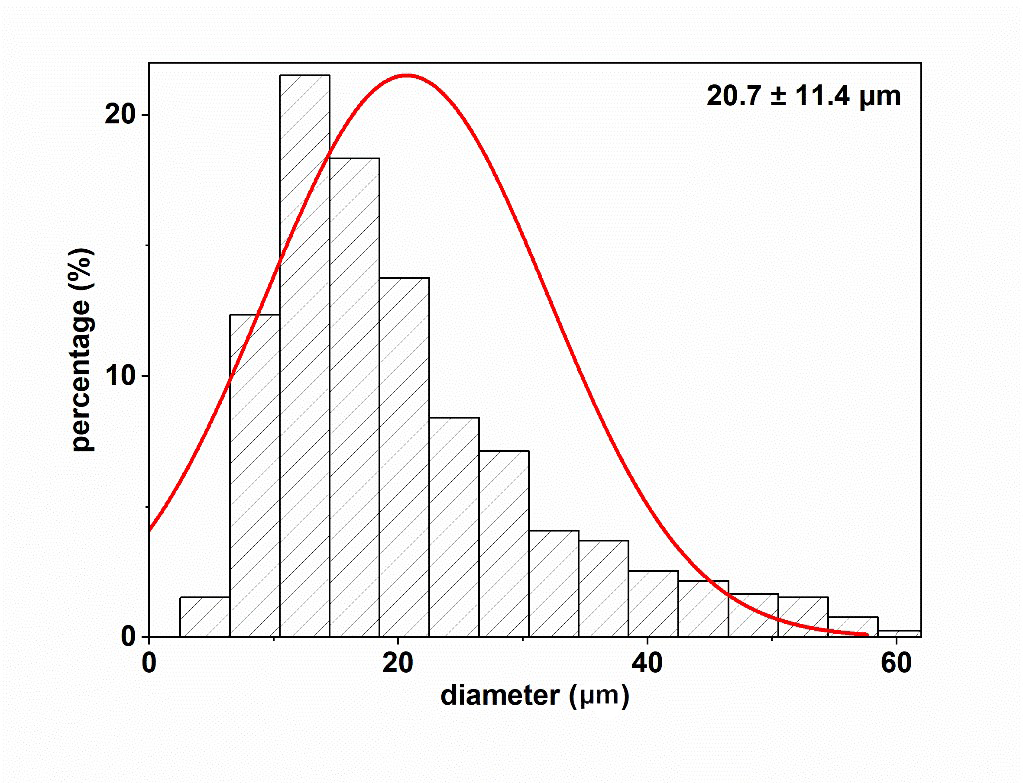


**Figure S8** Statistical diameter distribution of the microspheres achieved upon *ω* = 30 mg by SEM, showing a statistical average of 20.7 ± 11.4 μm. For accuracy, no less than 1000 records were counted for average.


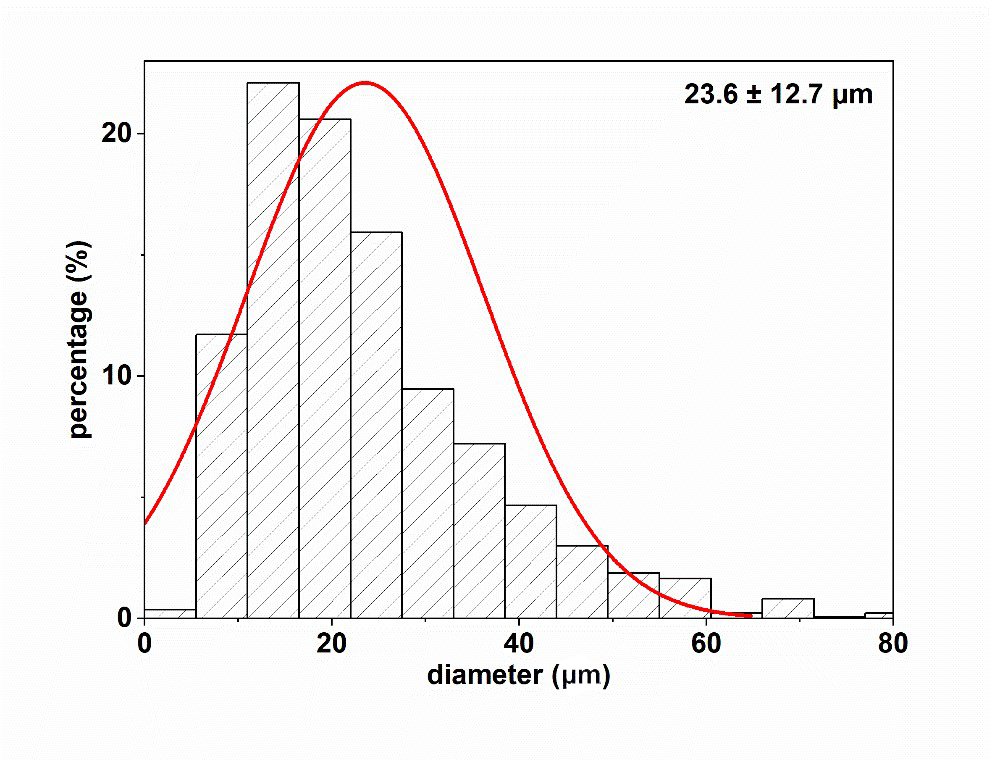


**Figure S9** Statistical diameter distribution of the microspheres achieved upon *ω* = 40 mg, showing a statistical average of 23.6 ± 12.7 μm. For accuracy, no less than 1000 records were counted for average.





**Figure S10** XRD patterns of the fibrillar and spherical architectures self-assembled by Fmoc-FF.

The results demonstrate that both the two supramolecular architectures showed a broad peak at 2θ = 19.2°, corresponding to a periodic spacing of 4.5 Å inside the self-assemblies, characteristic of the π-π stacking^1^.


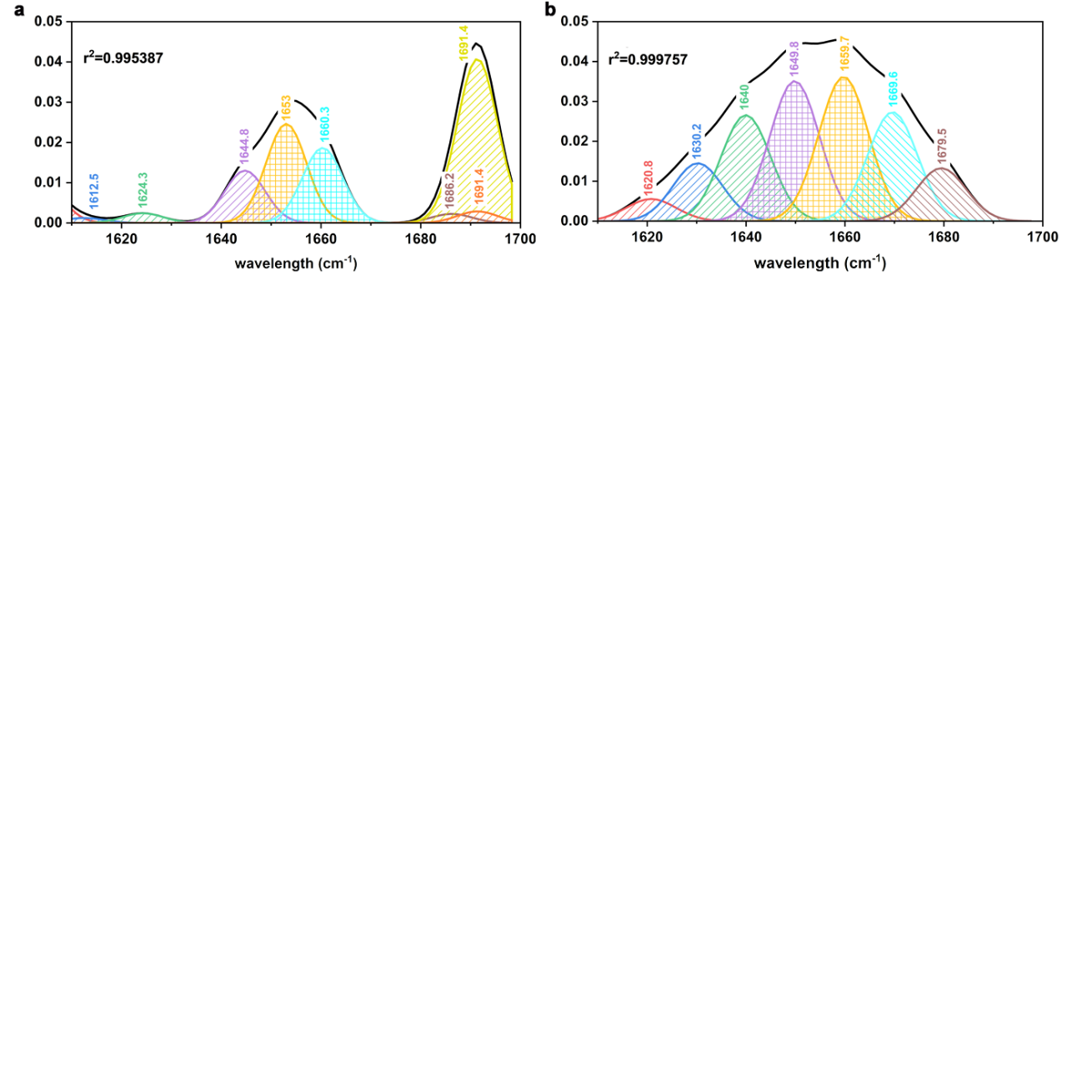


**Figure S11** Deconvolution analysis of the amide I region in the FTIR spectra of (**a**) fibrillar and (**b**) spherical architectures self-assembled by Fmoc-FF. The peak assignments are listed in Table S2.

**Table S2** Deconvoluted peak assignments in Figure S10.

| **No.** | **peak center (nm^-1^)** | **peak area** | **area sum** | **peak assignment^2^** |
| --- | --- | --- | --- | --- |
| **fibrillar architectures** | | | | |
| 1 | 1612.5 | 0.01268 | 0.17 | β-sheet |
| 2 | 1624.3 | 0.024696 |  | β-sheet |
| 3 | 1644.8 | 0.132394 |  | β-sheet |
| 4 | 1653.0 | 0.250489 | 0.44 | α-helix |
| 5 | 1660.3 | 0.189496 |  | α-helix |
| 6 | 1686.2 | 0.023005 | 0.47 | β-sheet |
| 7 | 1691.4 | 0.414444 |  | β-sheet |
| 8 | 1691.4 | 0.029241 |  | β-sheet |
| **spherical architectures** | | | | |
| 1 | 1620.8 | 0.06999 | 0.44 | β-sheet |
| 2 | 1630.2 | 0.183091 |  | β-sheet |
| 2 | 1630.2 | 0.183091 |  | β-sheet |
| 4 | 1649.8 | 0.442555 | 0.90 | α-helix |
| 5 | 1659.7 | 0.4561 |  | α-helix |
| 6 | 1669.6 | 0.344555 | 0.51 | β-turn |
| 7 | 1679.5 | 0.167443 |  | β-turn |

Tip: the statistical data are listed in Figure 3c in the main text for comparison.


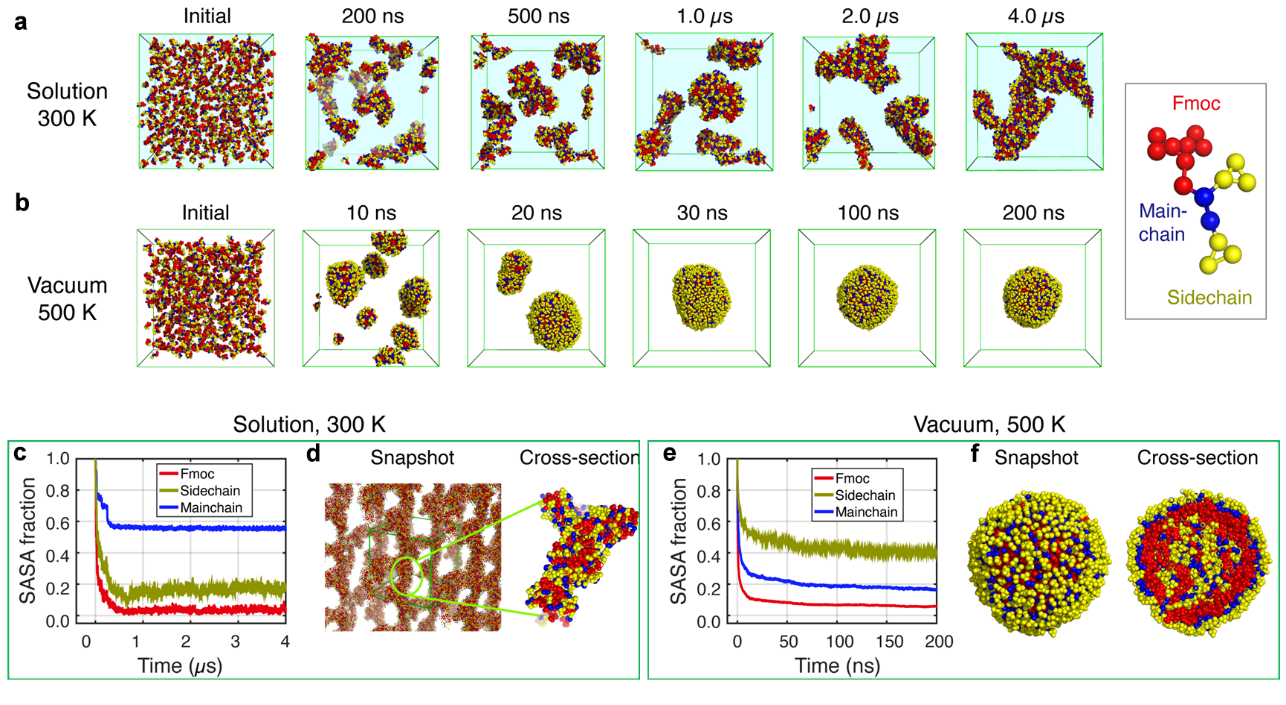


**Figure S12** Additional simulations on the self-assembly of Fmoc-FF in the solution phase and in the gaseous phase. (**a, b**) Snapshots at six time points showing Fmoc-FF forming (**a**) fibrillar aggregates in solution at 300 K (second-round of simulation with temperate the same as that in the main text) and (**b**) spherical assemblies in the gaseous phase at 500 K. (**c**) Time evolution of the SASA fraction for Fmoc, side-chain, and main-chain groups in solution, relative to their initial randomly dispersed states. (**d**) Snapshot and cross-sectional view of the fibrillar aggregate at the presence of solvents. (**e, f**) Similar to (**c, d**) but for the spherical aggregate in the gaseous phase.


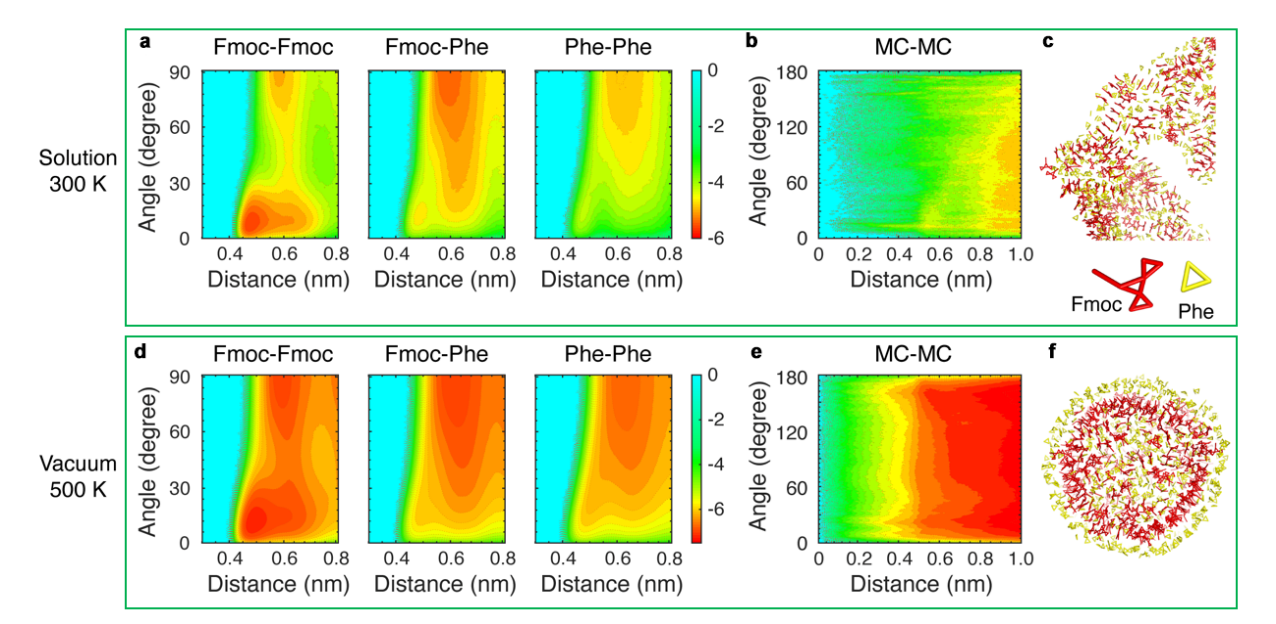


**Figure S13** FES analysis of Fmoc-FF self-assembly by additional simulations. (**a-c**) Fibrillar aggregation in the solvents at 300 K (second-round of simulation with temperate the same as that in the main text); (**d-f**) Spherical aggregation in the gaseous phase at 500 K. (**a, d**) FES of Fmoc-Fmoc, Fmoc-Phe, and Phe-Phe aromatic stacking and (**b, e**) MC-MC interactions as a function of centroid distance and angle between two aromatic rings/MCs for (top panel) fibril-like and (lower panel) spherical aggregates, respectively. (**c, f**) Cross-sectional views of the Fmoc-FF fibrillar and spherical aggregates, respectively, with Fmoc and Phe groups shown in stick representations.





**Figure S14** Typical force-distance profile for Young’s modulus measurement of Fmoc-FF self-assembled spheres arraying film in the gaseous phase.





**Figure S15** Statistical distribution of the Young’s modulus for spherical architectures achieved upon *ω* = 10 mg of Fmoc-FF, showing a statistical average of 100.5 ± 44.7 GPa.





**Figure S16** Statistical distribution of the Young’s modulus for spherical architectures deposited by 15 mg Fmoc-FF, showing the statistical average of 91.7 ± 49.9 GPa.





**Figure S17** Statistical distribution of the Young’s modulus for spherical architectures deposited by 40 mg Fmoc-FF, showing the statistical average of 77.5 ± 42.6 GPa.





**Figure S18** Statistical distribution of the Young’s modulus for spherical architectures deposited by 60 mg Fmoc-FF, showing the statistical average of 35.6 ± 30.5 GPa.





**Figure S19** Statistical distribution of the Young’s modulus for spherical architectures deposited by 80 mg Fmoc-FF, showing the statistical average of 18.1 ± 12.9 GPa.





**Figure S20** Normalized UV-vis absorption spectrum of the Fmoc-FF self-assembled spheres arraying film. The profile can be used to determine the Tauc plot for semiconductivity analysis.





**Figure S21** XPS spectrum of the Fmoc-FF self-assembled spheres arraying film. The valence band energy *vs.* Fermi level (*E_vbm_*) of the sample was analyzed by using Al Ka (1486.6 eV) as the X-ray source.





**Figure S22** UPS spectrum of the Fmoc-FF self-assembled spheres arraying film. The representative secondary electron cutoff region for the work function (*WF*) of the Fmoc-FF sample was analyzed using He I (21.2 eV) as the source.

**Table S3** Fitted parameters for the equivalent circuit shown in the inset of Figure 5g in the main text.

|  | C_g_ | R_i_ | R_e_ | C_dl_ | R_CT_ |
| --- | --- | --- | --- | --- | --- |
| in the dark | 67.0 nF | 1.40 Ω | 423.6 Ω | 2.30 μF | 2.2 GΩ |
| under irradiation^a^ | 1.3 μF | 1.47 Ω | 36.6 Ω | 55.0 μF | 33.6 kΩ |

*^a^* irradiation parameters: *λ* = 635 nm, 90 mW.


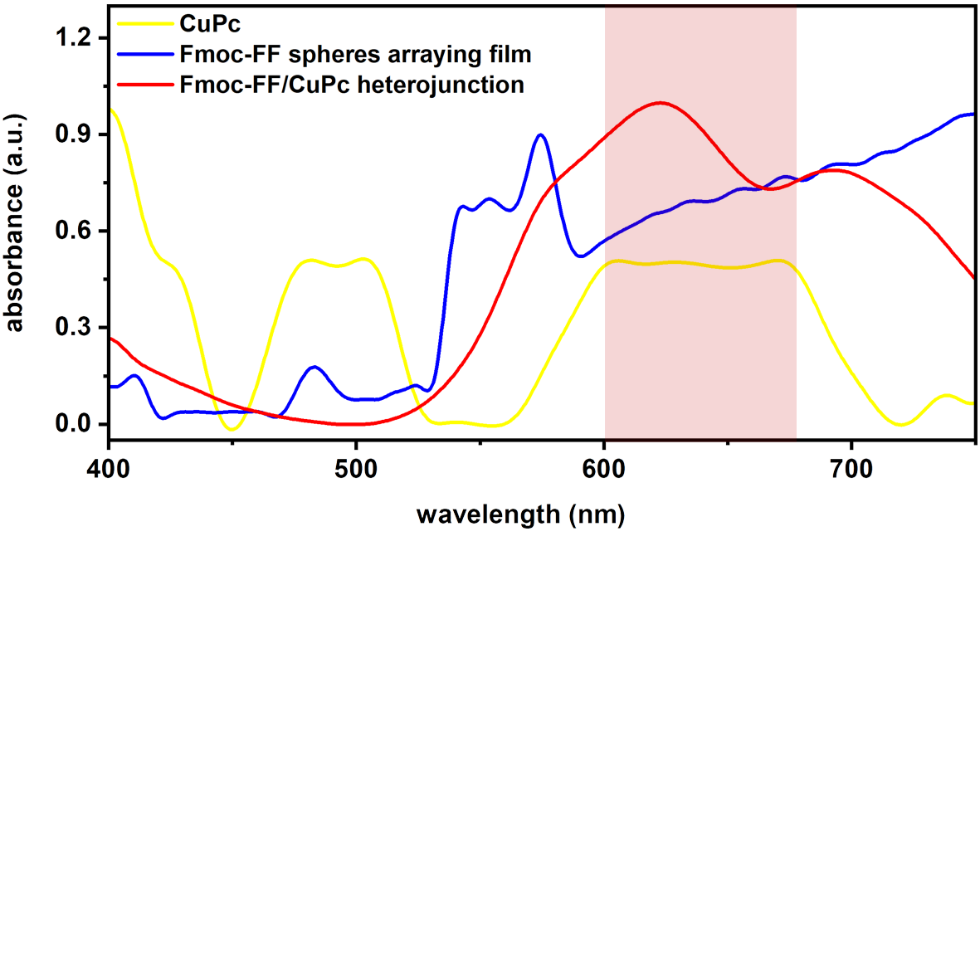


**Figure S23** UV-vis absorption spectra of the Fmoc-FF spheres arraying film, CuPc and Fmoc-FF/CuPc P-N heterojunctions.

The results demonstrate that both the P-type and N-type semiconductors studied herein showed strong adsorption in the range of 600-675 nm, suggesting the availability of photo-stimulated electrons transfer using the long-wavelength irradiation. Therefore, red light with a wavelength of 635 nm was utilized in the study to motivate the P-N heterojunctions.





**Figure S24** CV curve of the Fmoc-FF/CuPc P-N heterojunction-based photocapacitor from -0.5 to 1.2 V at various sweeping speeds.

The results demonstrate pseudo-rectangular voltammetry shape of the device, characteristic of the double-layer capacitance nature^3^.


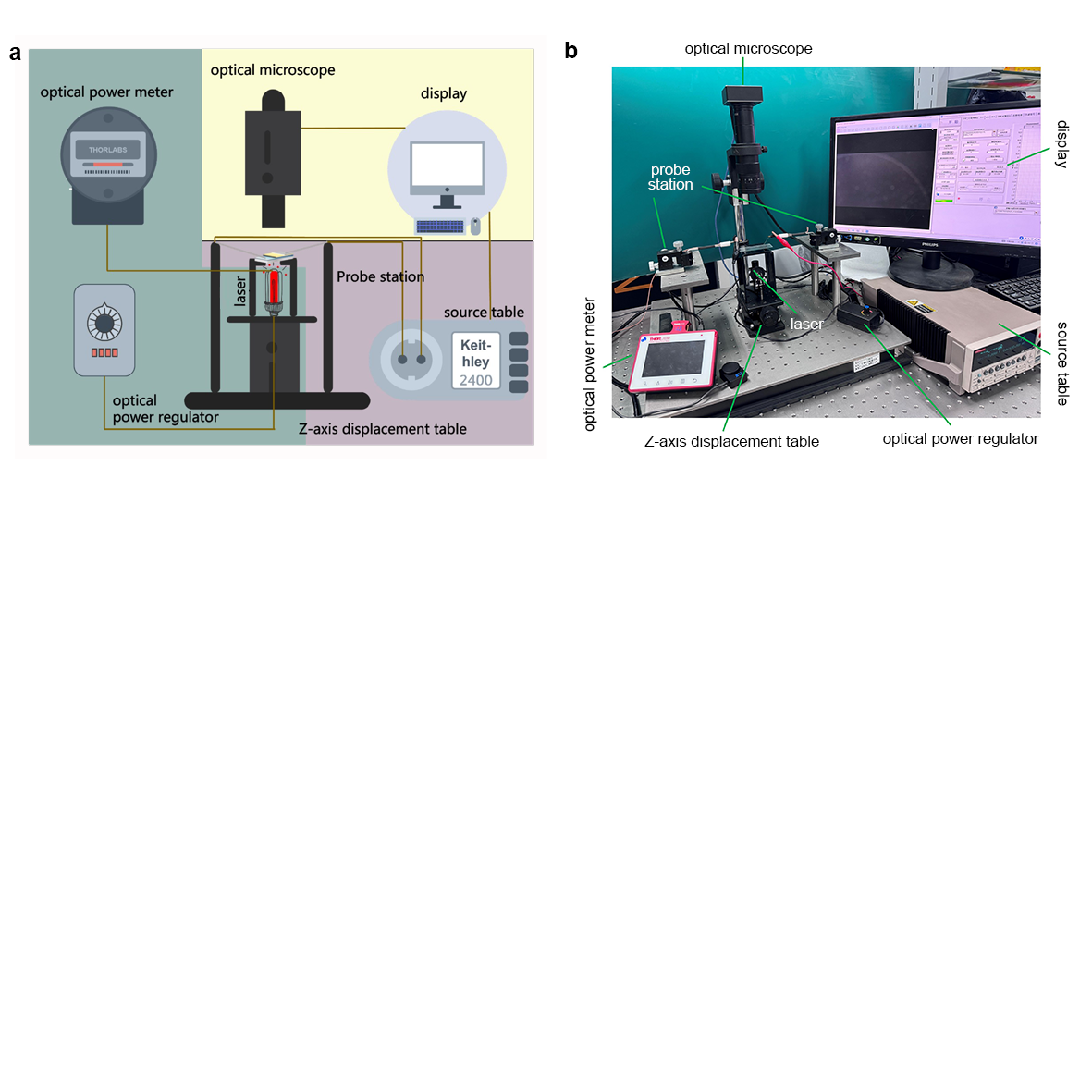


**Figure S25** Custom-designed photoelectronic testing system. (**a**) Schematic diagram and (**b**) Photographic picture of the setup.

**
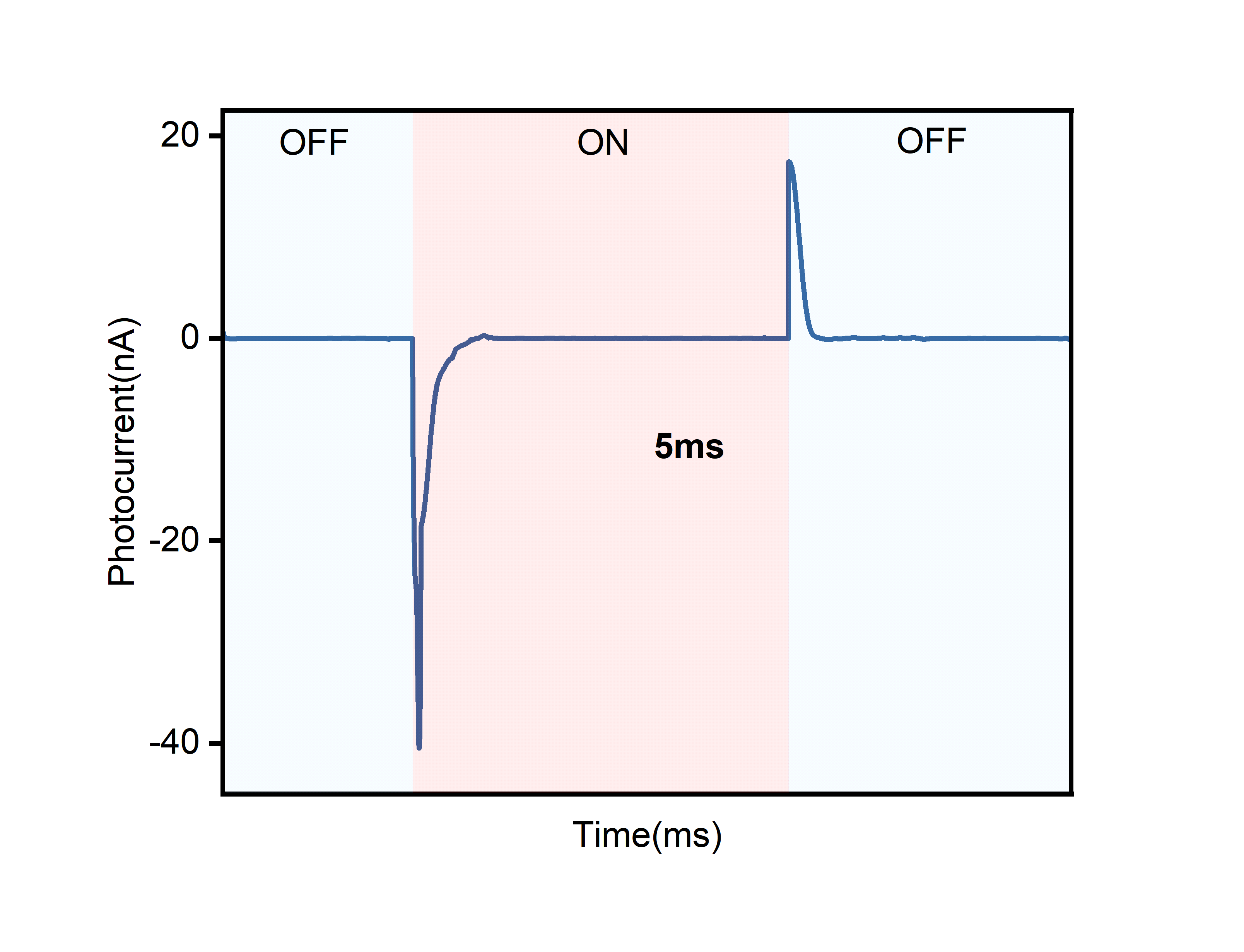
**

**Figure S26** Transient photocurrent measurement of CuPc alone-based photocapacitor under 5 ms light pulse stimulation.


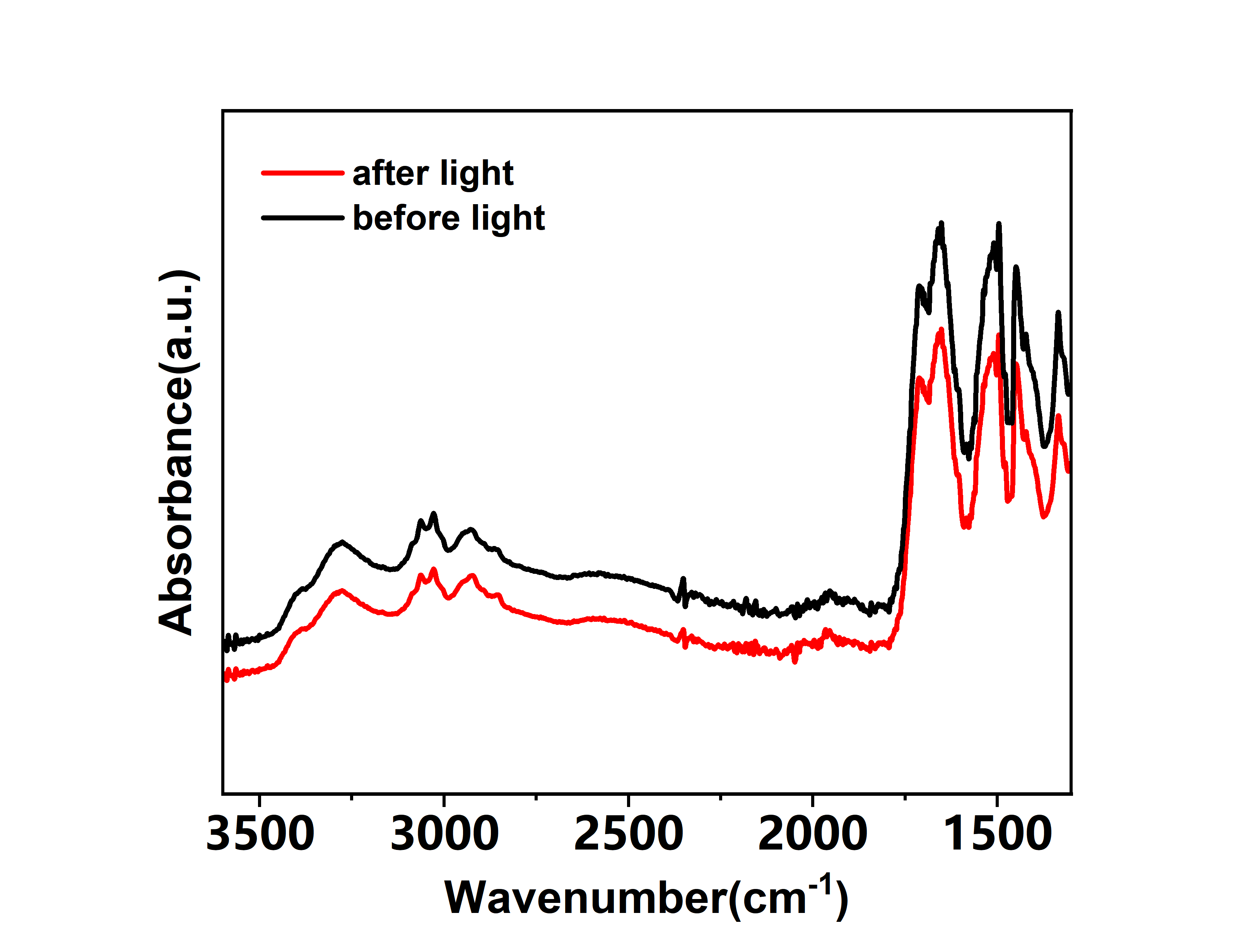


**Figure S27** FTIR spectra of the device before and after illumination.

**References**

[1] S. K. Burley, G. A. Petsko, *Science* **1985**, 229, 23.

[2] J. M. Bier, C. J. R. Verbeek, M. C. Lay, *Journal of Applied Polymer Science* **2013**, 130, 359.

[3] L. Adler-Abramovich, D. Aronov, P. Beker, M. Yevnin, S. Stempler, L. Buzhansky, G. Rosenman, E. Gazit, *Nature Nanotechnology* **2009**, 4, 849.
